# Structural Organization of S516 Group I Introns in Myxomycetes

**DOI:** 10.3390/genes13060944

**Published:** 2022-05-25

**Authors:** Betty M. N. Furulund, Bård O. Karlsen, Igor Babiak, Peik Haugen, Steinar D. Johansen

**Affiliations:** 1 Genomic Division, Faculty of Biosciences and Aquaculture, Nord University, 8049 Bodø, Norway; betty.martine.furulund@nordlandssykehuset.no (B.M.N.F.); igor.s.babiak@nord.no (I.B.); 2 Research Laboratory and Department of Microbiology, Nordland Hospital Trust, 8005 Bodø, Norway; bard.ove.karlsen@nordlandssykehuset.no; 3 Department of Chemistry and Center for Bioinformatics, Faculty of Science and Technology, UIT—The Arctic University of Norway, 9037 Tromsø, Norway; peik.haugen@uit.no

**Keywords:** catalytic introns, homing endonuclease, intron evolution, ribozyme, twintron

## Abstract

Group I introns are mobile genetic elements encoding self-splicing ribozymes. Group I introns in nuclear genes are restricted to ribosomal DNA of eukaryotic microorganisms. For example, the myxomycetes, which represent a distinct protist phylum with a unique life strategy, are rich in nucleolar group I introns. We analyzed and compared 75 group I introns at position 516 in the small subunit ribosomal DNA from diverse and distantly related myxomycete taxa. A consensus secondary structure revealed a conserved group IC1 ribozyme core, but with a surprising RNA sequence complexity in the peripheral regions. Five S516 group I introns possess a twintron organization, where a His-Cys homing endonuclease gene insertion was interrupted by a small spliceosomal intron. Eleven S516 introns contained direct repeat arrays with varying lengths of the repeated motif, a varying copy number, and different structural organizations. Phylogenetic analyses of S516 introns and the corresponding host genes revealed a complex inheritance pattern, with both vertical and horizontal transfers. Finally, we reconstructed the evolutionary history of S516 nucleolar group I introns from insertion of mobile-type introns at unoccupied cognate sites, through homing endonuclease gene degradation and loss, and finally to the complete loss of introns. We conclude that myxomycete S516 introns represent a family of genetic elements with surprisingly dynamic structures despite a common function in RNA self-splicing.

## 1. Introduction

Group I introns are self-splicing and mobile elements sporadically distributed within genomes of bacteria, bacteriophages, chloroplasts, mitochondria, eukaryotic viruses, and the nuclei of eukaryotes [1,2]. Nuclear group I introns are restricted to ribosomal DNA (rDNA) of fungi and protists and appear common in myxomycetes (plasmodial slime molds) [3]. Most nucleolar group I introns interrupt functionally important sequences in the small subunit (SSU) and large subunit (LSU) rRNAs corresponding to the peptidyl transferase center and decoding center [4,5]. Intriguingly, a given insertion site harbors related intron sequences with a common evolutionary history even in distantly related taxa [6,7,8,9,10,11]. An rDNA group I intron nomenclature has been established based on species name abbreviation, insertion site, and the *E. coli* rRNA gene numbering system [12].

Group I introns catalyze their own splicing due to the intron-encoded ribozyme. The splicing mechanism is well described and involves two succeeding transesterification reactions initiated by an attack of an exogenous guanosine (exoG) cofactor [3,13]. The resulting products are (i) a perfectly ligated exon RNA originating from transcribed host gene and (ii) the excised intron RNA sequence. Most nuclear group I introns also catalyze an exoG-independent reaction pathway leading to full-length intron circles and fragmented exons [14]. The catalytic RNA activities are dependent on a highly organized catalytic core structure composed of a series of paired segments (P1 to P9), which are further organized into three structural domains [3,15]. A crucial structural segment in catalysis is the P7 element, which is located at the center of the catalytic core [16]. Nuclear group I introns are restricted to either one of two subgroups, i.e., group IC1 and group IE, which possess unique and characteristic structural features [3,17,18,19].

Nuclear group I introns may also carry additional genes and sequences. Most common are homing endonuclease genes (HEGs), which are found in approximately 5–10% of known nuclear group I introns and code for site-specific endonucleases involved in intron mobility [3,20,21,22]. Nuclear HEGs are reported to be inserted into different peripheral intron segments in P1, P2, P6, P8, or P9, and the corresponding proteins belong to the His-Cys family of homing endonucleases [23,24]. Several structural features appear to facilitate the cellular expression of nuclear HEGs embedded in ribosomal DNA [25]. Some homing endonuclease mRNAs have a unique lariat cap at their 5′ ends catalyzed by a separate intron-encoded ribozyme [26,27,28], and most appear polyadenylated at their 3′ ends [11,29,30]. Interestingly, several HEGs also carry small spliceosomal introns of about 50 nt that have to be removed in order to generate a functional open reading frame [11,29,30,31] and thus may facilitate the expression.

Several examples of direct repeat (DR) arrays within nuclear group I introns have been reported [7,9,11,32,33]. These arrays appear to have a similar location in the intron RNA structure as HEGs, but their biological role remains unknown. Variations in sequence motifs, number of copies, and size of arrays are generally observed, but a common feature appears that arrays do not interfere with ribozyme functions due to their locations in peripheral segments. Recently, we reported a complex pattern of DR array within the S1389 group I intron in the myxomycete *Mucilago crustacea* that contained 34 copies of the intron internal guide sequence [11], which may suggest a functional role in splicing.

In a previous study, we reported self-splicing and structural organization of nuclear S516 group I introns [6]. These introns interrupt a tRNA binding and decoding domain of the SSU rRNA [34] and have to be precisely removed by splicing in order to restore a functional rRNA. Introns at position S516 were found in ascomycete and basidiomycete fungi, and in various protists phyla such as the red, green, and brown alga, in amoeba and amoeba-flagellates, and in myxomycetes. Some of the introns belonged to the group IE subclass (e.g., ascomycetes and green alga) whereas others to the group IC1 subclass (e.g., red alga, amoeba, amoeba-flagellates, and myxomycetes). The group IC1 introns showed the most variability in structural organization, and HEGs and pseudo-HEGs were identified in red alga [35,36], in amoeba [6], and in amoeba-flagellates [37]. The latter group involved twin-ribozyme introns in several *Naegleria* species and in *Allovahlkamfia* and harbored complex insertions in the P6 segment corresponding to HEGs and lariat-capping ribozymes [28,38,39].

In the original S516 intron study [6], only three myxomycete introns were available. These introns were from related taxa, had a similar RNA structural fold, and contained no HEG insertions. In the present study, we extended the analysis to include 75 S516 group IC1 introns in myxomycetes representing five taxonomic orders. The intron RNA possessed a conserved catalytic core region, but highly variable peripheral regions due to DR arrays and HEGs interrupted with small spliceosomal introns.

## 2. Materials and Methods

### 2.1. Small Subunit Ribosomal DNA Sequences

All rDNA sequences included in this work, except that of *Didymium alpinum* isolate Fr-K12, were retrieved from the NCBI database (https://www.ncbi.nlm.nih.gov, accessed on 15 March 2022; Table S1). *D. alpinum* was collected as spores in the French Alps by Ms. Kari Haugli (University of Tromsø, Tromsø, Norway). Spores were germinated and approximately 10^8^ amoeba cells were harvested from DS/2 agar plates, total DNA extracted, and rDNA sequenced as described previously [8].

### 2.2. Sequence Alignments

SSU rDNA sequences (with introns removed) were assembled and aligned in the software program Geneious prime^®^ 2022.0.1 (https://www.geneious.com, accessed on 15 February 2022). Multiple sequence alignments were generated using MAFFT version 7.450 [40,41] with default settings. The SSU rDNA dataset 1 (1524 nt) and dataset 2 (1784 nt) alignments were manually adjusted according to published information about myxomycete SSU rDNA genes [42,43], excluding parts of variable regions that were not confidently aligned. Group I intron core sequences (143 bp) were manually aligned and strictly based on secondary structure features as described previously [6,8,11] using the Geneious prime^®^ software program.

### 2.3. Phylogenetic Analysis

The tree-building methods of neighbor joining (NJ), maximum likelihood (ML), maximum parsimony (MP), and minimal evolution (ME), interpreted in MEGA X [44], were used in the SSU rDNA and S516 intron datasets with default settings as reported previously [11]. All sequence alignments were model tested prior to tree constructions by the MEGA X software [44]. The topology of NJ, ML, and ME trees was evaluated by bootstrap analyses. The evolutionary relationship generated by SSU rDNA dataset 1 was reconstructed with NJ and ML using the Kimura 2 (K2) evolutionary model [45]. The robustness was tested by bootstrapping (500 replicates). SSU rDNA dataset 2 was reconstructed with NJ, ML, and ME using the K2 evolutionary model [45]. The robustness was tested by bootstrapping (500 replicates). The evolutionary relationships of S516 intron core sequences (intron dataset 1) were reconstructed with the NJ method and the Jukes–Cantor (JC) model using MEGA X [44] with default settings. To test the robustness of the nodes, the trees were tested with NJ-JC (500 replicates) and ME-JC (500 replicates). Intron dataset 2 was reconstructed with NJ, ML, and ME using the JC evolutionary model [45]. The robustness was tested by bootstrapping (500 replicates).

## 3. Results

### 3.1. Diderma alpinum Fr-K12 Contains a Group I Intron at Position 516 in the Nuclear SSU rRNA Gene

**Figure 1:**
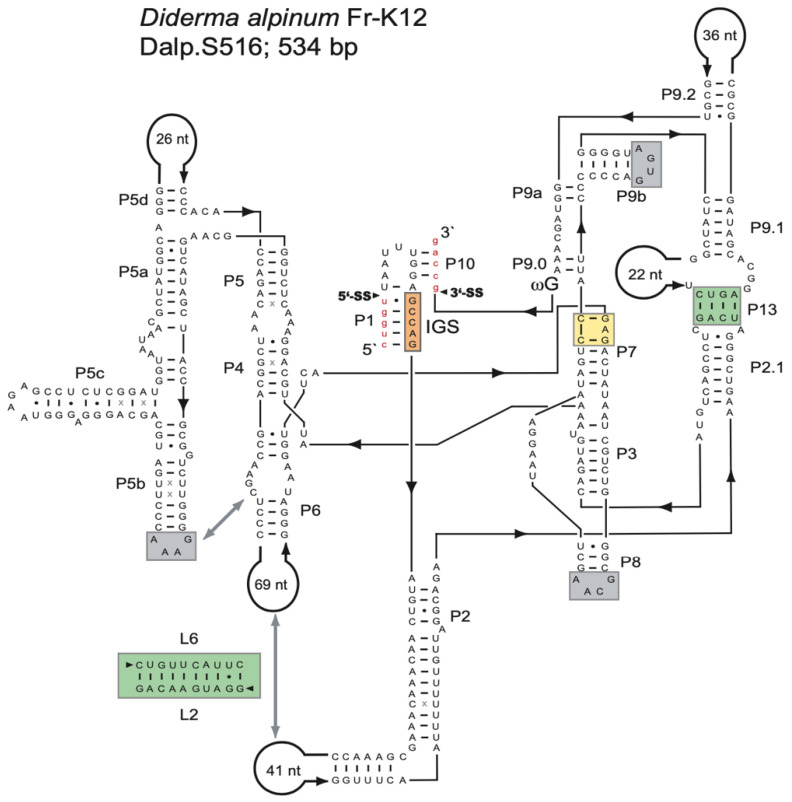
Secondary structure of the *Diderma alpinum* S516 group I intron RNA. The diagram of the representative 534-nt group IC1 intron RNA is presented according to [15]. The intron has a well-conserved core organization based on paired RNA segments (P1-P10). The guanosine binding site in P7 is indicated (yellow box), as well as the universal conserved terminal guanosine (ωG). Long-distance RNA–RNA interactions are commonly found in group I intron RNAs, involving receptors and GNRA-loops (gray boxes), or regular base pairings (green boxes). 5′SS and 3′SS, exon-intron splice sites; IGS, internal guide sequence: (-) Watson–Crick base pair; (•) non-Watson–Crick GU base pairing; (x) additional non-Watson–Crick base pairings with comparative support. Exon sequences are shown as red lowercase letters.

Ribosomal DNA sequence analysis of the myxomycete *D. alpinum* (Fr-K12) identified a 534-bp group I intron (Dalp.S516) at position S516 in the nuclear SSU rRNA gene. An RNA secondary structure diagram is presented in Figure 1. Dalp.S516 represents a typical group IC1 intron fold, similar to that of the *Tetrahymena* intron [19,46,47], with a well-conserved core organization based on paired RNA segments (P1–P10). Several general structure features common to group IC1 introns are present (Figure 1): (i) A conserved U:G pair at the 5′SS in segment P1, which partly constitutes the internal guide sequence and is critical in the first reaction step of intron self-splicing. (ii) A conserved guanosine binding site in segment P7 (yellow box) is essential in the catalytic site of the ribozyme. (iii) A tertiary segment (P13) that likely contributes to the overall stability of the ribozyme core (green box). (iv) Three GNRA tetra-loops (within the P5b, P8, and P9b extensions) were probably also involved in RNA–RNA interactions and stability. The GAAA-loop in P5b is expected to interact with a predicted receptor in P6. (v) A conserved terminal guanosine (ωG) at the 3′SS is involved in the second reaction step of catalysis. (vi) A proposed and putative 10-bp interaction between P2 and P6 extensions, with a possible role in ribozyme core stability (green box). It is important to note that this interaction has not been tested experimentally.

### 3.2. Structure Variation among Myxomycetes S516 Group I Introns

**Figure 2:**
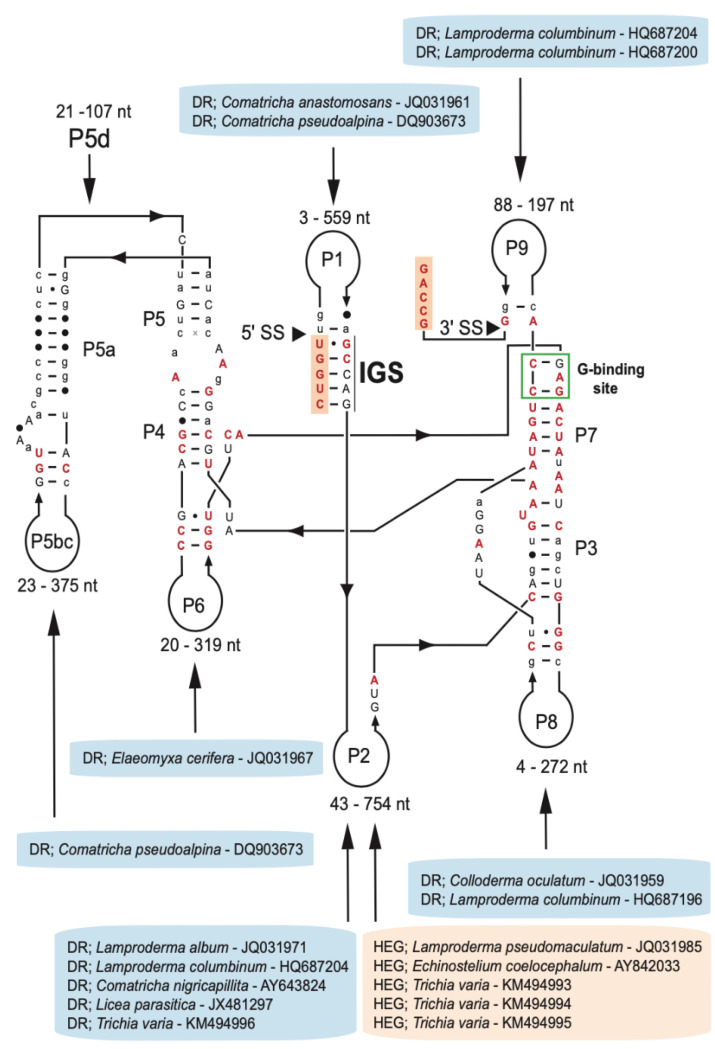
Consensus secondary structure diagram of S516 group I intron RNA in myxomycetes. The consensus structure is based on 143 nucleotide positions in the catalytic core common among introns from 75 taxa (see Table S1). Extensive sequence size variations are noted in peripheral regions. While all homing endonuclease genes (HEGs) are found as P2 extensions, direct repeat (DR) extensions are located in most peripheral regions. The guanosine (G) binding site in P7 is indicated. P1–P9, paired RNA segments; 5′SS and 3′SS, exon-intron splice sites. Invariant nucleotide positions among the 75 introns are shown as red uppercase letters. Black uppercase letter, ≥90% conservation; lowercase letters, ≥50% conservation; filled circles, <50% conservation.

Group I introns at position S516 are widely but sporadically distributed among fungi and protists, including myxomycetes [6]. We collected 75 myxomycete S516 introns representing five distantly related orders (Physarales, Stemonitales, Liceales, Echinosteliales, and Trichiales), 21 genera, and 38 distinct species (Table S1). The majority of the SSU rRNA gene sequences were retrieved from the NCBI database, and one sequence (*D. alpinum* Fr-K12) was provided in this study. To assess structural variations among the S516 introns, 143 nucleotide positions in the ribozyme core region common to all 75 introns were strictly aligned according to secondary structure features (Figure S1). The consensus secondary structure diagram (Figure 2) shows a typical group IC1 intron fold with a conserved substrate (P1–P2), scaffold (P4–P5–P6), and catalytic (P3–P7–P8) domains [see 19,46]. They all show a conserved guanosine binding site in P7, an extended P5d hinge region, and a P13 segment. The latter represents a common but only weakly conserved structural feature among the S516 introns. Most sequence variations are found in their peripheral extension regions, especially the P2 extension. Here, five S516 introns contain homing endonuclease gene (HEG) insertions, and eleven S516 introns were found to contain direct repeat (DR) arrays. HEGs were exclusively identified in P2, whereas DRs were found in all extension regions (Figure 2).

### 3.3. Complex Inheritance Pattern of Myxomycete S516 Group I Introns

Evolutionary relationships among the myxomycete S516 introns were assessed by phylogenetic analyses. First, a host gene phylogeny was established based on an alignment of SSU rDNA sequences covering 1524 positions from 66 taxa. The topology of a representative neighbor-joining (NJ) tree (Figure S2) is in general agreement with previously reported studies using a similar selection of myxomycete taxa [11,33,48]. Next, we compiled an intron dataset containing 143 sequence positions from the catalytic core region from a total of 75 myxomycete S516 group I introns (Figure S1) and inferred the evolutionary relationships using the NJ method (Figure S3). Whereas some of the clades are strongly supported by bootstrap analyses, the positioning of the same clades in the overall tree topology is not. As noted earlier for S1389 group I introns [11], intron-family phylogenies are typically based on few aligned positions and a large number of taxa, thus resulting in trees with limited robustness of nodes. We did, however, observe an overall congruency between the SSU rDNA and intron-based phylogenies, particularly between clades with closely related introns (e.g., among *Diderma* taxa and among *Trichia* taxa; compare Figures S2 and S3), suggesting that at least some S516 group I introns possessed a vertical inheritance pattern during the evolution of myxomycetes.

**Figure 3:**
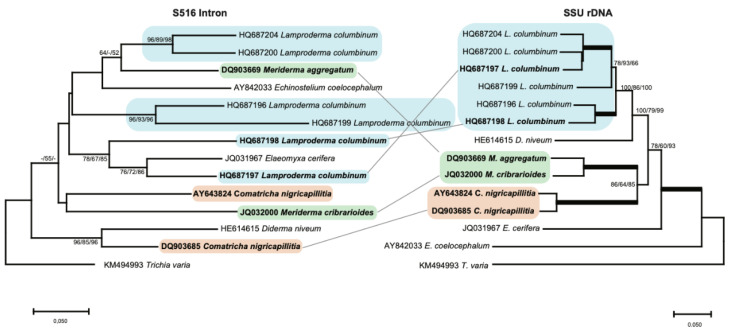
Molecular phylogeny of S516 group I introns as candidates to horizontal transfer. The intron and SSU topology is obtained by neighbor-joining (NJ) analysis of 14 taxa and 143-nt aligned positions (intron dataset 2; Table S1) and 1784-nt aligned positions (SSU rDNA dataset 2; Table S1). The NJ, maximum likelihood (ML), and minimal evolution (ME) bootstrap replicates (≥50%) are given for each node. Isolates observed as horizontal inheritance candidates are shown in bold letters. Bold branches in the SSU rDNA tree indicate maximum support in NJ (≥98%), ML, and ME (≥98%). The scale bar indicates the fraction of substitutions per site.

Most introns, however, showed a relationship that is not consistent with stable vertical inheritance. To better resolve the evolutionary relationship between S516 introns, we analyzed a smaller dataset consisting of 14 selected taxa (Figure 3), and from the resulting NJ tree, we made several interesting observations. (i) While the six *L. columbinum* isolates cluster together in the SSU rDNA analysis to a well-defined clade (blue box), the corresponding introns are found scattered on the tree, e.g., the *L. columbinum* (HQ687198) and *L. columbinum* (HQ687197) introns are distantly related to the other *L. columbinum* introns. (ii) SSU rDNA analysis strongly support that *M. aggregatum* and *M. cribrariodes* are two closely related taxa from the same genus (green box), whereas intron analysis of the core region indicates that the S516 introns are distantly related. This is further supported by structural comparisons (Figure S4), where P2 and P6 peripheral extensions were found to be clearly different. (iii) Two closely related *C. nigricapillitia* isolates cluster together in the SSU rDNA analysis (orange box), but their corresponding S516 introns suggest a distant relationship. In summary, our phylogenetic analyses suggest that myxomycete S516 introns share a complex evolutionary history, with several clear examples of both vertical and horizontal transfers.

### 3.4. Spliceosomal Introns Interrupt Homing Endonuclease Genes of S516 Group I Intron

**Figure 4:**
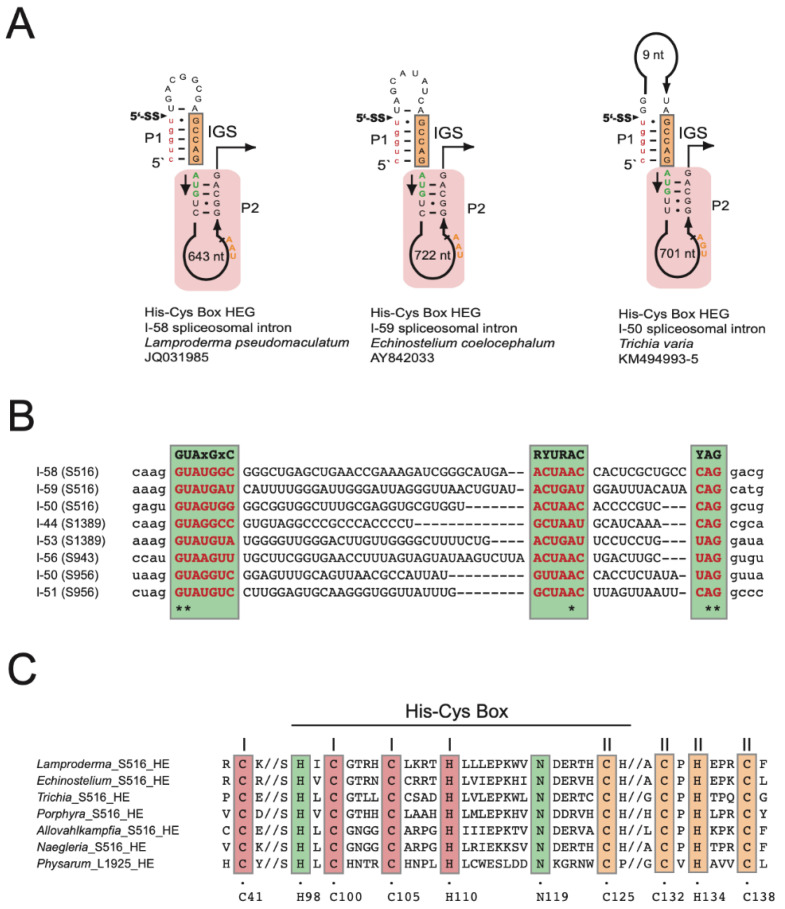
His-Cys Box homing endonucleases encoded by S516 group I introns. (**A**) Schematic organization of the S516 intron substrate domain hosting homing endonuclease genes (HEGs) in P2. 5′SS, exon-intron splice site; IGS, internal guide sequence. The HEG start codons (AUG) and stop codons (UAA/UGA) are indicated. HEGs are interrupted by small spliceosomal introns (I-50, I-58, and I-59). (**B**) Sequence alignment of spliceosomal introns (uppercase letters) with some flanking HEG sequences (lowercase letters). Sequence motifs common to the mammalian spliceosomal intron consensus are indicated (green boxes), and invariable positions at the 5′SS (GU), branch site (A), and 3′ splice site (AG) are indicated by * below the alignment. (**C**) Amino acid alignment of His-Cys Box features of S516 homing endonucleases (HE). The *Naegleria* and *Physarum* HEs represent well-studied and experimentally verified His-Cys homing endonucleases. Conserved residues (boxed) corresponding to those presented in the *Physarum* L1925 HE crystal structure [52]. C41, C100, C105, and H110 are involved in zinc-binding motif I. C125, C132, H134, and C138 are involved in zinc-binding motif II. H98 and N119 are associated with the active site.

The S516 HEGs are organized in sense orientation compared to the intron ribozymes and host SSU rDNA. Moreover, the HEGs are located only in the intron P2 segment (Figure 4A) and encode His-Cys homing endonucleases of 187 amino acids in *L. pseudomaculatum* (JQ031985) (Figure S5), 190 amino acids in *E. coelocephalum* (AY842033) (Figure S6), and 210 amino acids in *T. varia* (KM494993/4/5) (Figure S7). Furthermore, the S516 HEGs appear sporadically distributed among myxomycetes except for *T. varia*. Here, the three HEG-containing isolates cluster together on the host phylogeny with one *T. varia* isolate (KM494996) lacking a HEG insertion (Figure S2). Interestingly, all S516 HEGs are interrupted by small spliceosomal introns of either 58 bp, 59 bp, or 50 bp (Figure 4A). A nucleotide sequence alignment of the spliceosomal introns from four different SSU rDNA group I intron sites (S516, S943, S956, and S1389) show that the introns contain well-conserved splice sites and branch sites, which places them in the GT-AG class of spliceosomal introns (Figure 4B).

A hallmark of nuclear homing endonucleases is a His-Cys box motif consisting of two zinc coordinating structures (Zn-I and Zn-II) and two catalytic amino acid residues. Figure 4C shows that the His-Cys box motif in myxomycete S516 introns is highly conserved when compared to S516 homing endonucleases in *Porphyra* (red algae) [49], *Allovahlkampfia* (amoeba-flagellate) [39], and *Naegleria* (amoeba-flagellate) [50], as well as the structurally characterized I-*Ppo* I from the myxomycete *Physarum polycephalum* [51,52].

### 3.5. Direct Repeat Arrays in Peripheral Extensions of S516 Group I Introns

DR features were found in eleven S516 introns representing six myxomycete genera (*Elaeomyxa*, *Comatricha*, *Colloderma*, *Lamproderma*, *Licea*, and *Trichia*), and all except two (*Licea* and *Trichia*) classified to the myxomycete order Stemonitales (Table S1). According to the consensus diagram (Figure 2), DRs were located within six peripheral extension regions (P1, P2, P5, P6, P8, and P9), all of which do not structurally interfere with the catalytic core. A closer inspection of DR sequences revealed a high degree of sequence heterogeneity in sequence motif, sequence length, and copy number (Table S2). Sequence heterogeneity between individual motifs within the same array was common (Figure S8).

**Figure 5:**
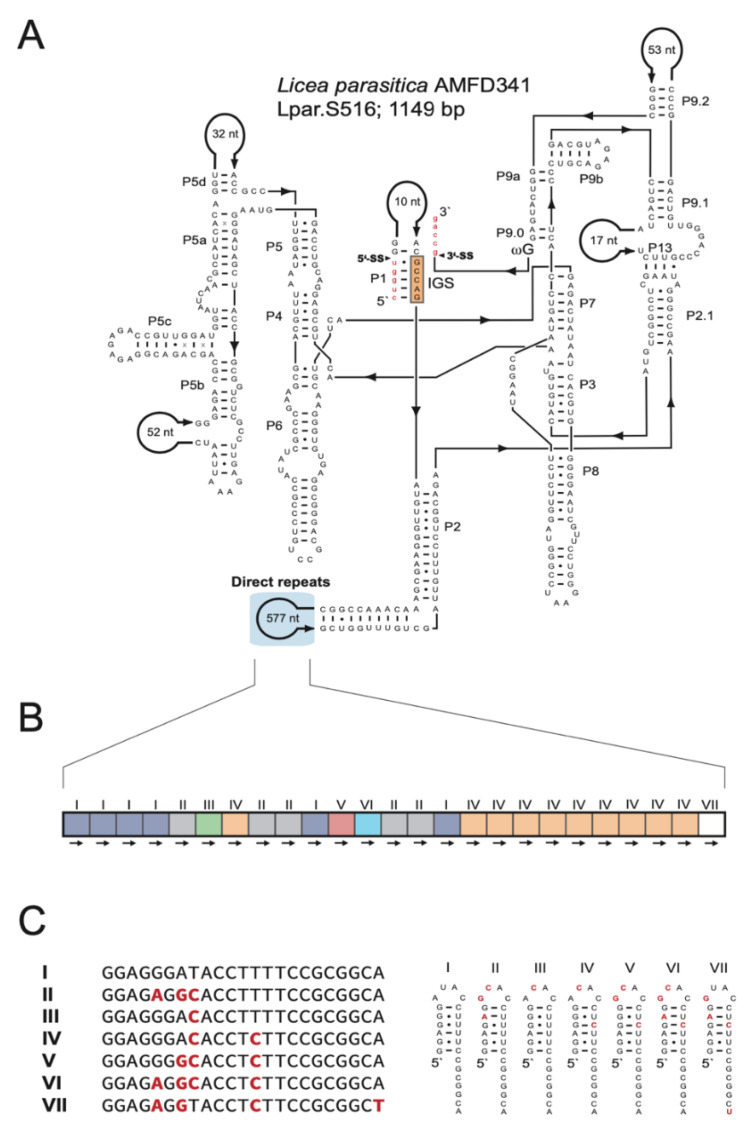
Direct repeat arrays in peripheral regions. (**A**) Secondary structural diagram of the S516 group I intron RNA in *Licea parasitica* containing direct repeats in the P2 peripheral region. For structural annotations, see legend to Figure 1. (**B**) Schematic organization of the direct repeats, which consists of 25 copies and seven motif variants (I to VII). (**C**) Alignment of sequence variants (left) and possible RNA hairpin structure formation (right).

Some isolates showed unique and characteristic DR features (Figure S8): (i) *L. album* (JQ031971) harbors the largest motif (ca. 220 nt), which is located in P2 and repeated twice. (ii) *C. nigricapillitia* (AY643824) harbors the smallest motif (only 4 nt) in P2 and is repeated four times. (iii) *C. pseudoalpina* (DQ903673) harbors DRs in two different extension regions (P1 and P5). While the P1-DR motif is large in size (ca. 110 nt) and repeated four times, the P5-DR is composed of an approximately 60-nt motif and repeated twice. (iv) *L. columbinum* (HQ687204) also harbors DRs in two extension regions, but in P2 and P9. Two DR motifs are located in P2 (ca. 20 nt and ca. 40 nt), in 5 and 3 copies, respectively, and the DR motif in P9 is 28 nt in size and repeated twice. (v) *E. cerifera* (JQ031967) harbors three different DR motifs in P6 of 11 nt, 10 nt, and ca. 80 nt in sizes, repeated 4, 6, and 2 times, respectively. (vi) One each of two closely related *T. varia* introns contains (KM494995; 1145 bp) or lacks (KM494996; 482 bp) a HEG. Intriguingly, the shorter variant contains a DR, including a HEG remnant sequence, at the proposed HEG deletion site (Figure S9). (vii) Finally, *L. parasitica* (JX481297) harbors a complex array in P2. Specifically, the intron folds into a typical group IC1 intron structure (Figure 5A), and the large size (1149 bp) is mainly due to a 577-nt extension in P2. No recognizable open reading frame was found, but P2 harbors a DR array with 25 copies of a 23-nt motif (Figure 5B). Seven motif variants (named I to VII) are present, and repeated either once (III, V, VI, and VII), five times (II), six times (I), or ten times (IV). Interestingly, each motif apparently folds into an RNA hairpin structure where heterogenic positions are either located within the single-stranded loop or present as compensatory base pairs in the stem region (Figure 5C).

## 4. Discussion

We report analyses of structural organization and molecular evolution of nucleolar group I intron at position S516 in myxomycetes. All 75 introns belong to the group IC1 subtype and fold at the RNA level into a highly conserved catalytic core, exemplified by the *D. alpinum* Fr-K12 intron. Extension sequences such as HEGs or DR arrays are all located in peripheral segments that do not interfere with the catalytic ribozyme core. HEGs located as insertions into segment P2 in five taxa are interrupted by small spliceosomal introns. Moreover, DR arrays were found in 11 taxa at six peripheral segments, and the *L. parasitica* intron was found to harbor a highly structured DR motif in 25 copies. Intron evolution analyses indicate a complex inheritance pattern that apparently includes both vertical and horizontal transfers.

All HEGs in myxomycete S516 introns encode His-Cys homing endonucleases with a characteristic set of conserved amino acid residues involved in zinc coordination and catalysis. This His-Cys box was found to be highly conserved among S516 HEGs, also outside the myxomycete phylum. These include the *Porphyra* red algae [49] and the *Allovahlkampfia* and *Naegleria* amoeba-flagellates [38,39], which support a common origin of S516 HEGs. The *Naegleria* homing endonucleases have been studied in more detail and found to recognize a 19 bp partially symmetrical sequence at the intron-less allele close to the S516 insertion site and to generate a pentanucleotide 3′ overhang at the DNA cleavage site [50,53]. The myxomycete S516 HEGs were all found to be interrupted by small spliceosomal introns similar to those found in several other group I intron HEGs at SSU rDNA positions S943, S956, and S1389 [11,29,30,31]. The presence of spliceosomal introns in HEGs is proposed to facilitate the expression of protein-coding genes embedded in nuclear rDNA [25].

Extension sequences containing DR arrays were noted in six peripheral segments, including P2. The arrays varied from small insertions (4-nt motif in 4 copies) in *C. nigricapillitia* to 23-nt motif in 25 copies in *L. parasitica*. A common feature to most arrays was non-identical sequence repeat motifs, which argues against slippage-like mechanisms during replication [54,55] as a cause of repeat origin and maintenance. Then, what could be the biological role, if any, of the DR arrays in nuclear group I introns? DR arrays have been reported in several nuclear group I introns in myxomycetes, especially in introns that are unable to self-splice in vitro [7,9,11,33]. In *Fuligo septica* L1949 [7] and *M. crustacea* S1389 [11], intron DR arrays resulted in multiple alternative P1 segments (carrying the 5′ splice site), suggesting that DRs potentially can interfere with intron splicing. Myxomycete S516 introns, however, appear to self-splice efficiently as naked RNA in vitro [6]. The highly structured hairpins with compensatory nucleotide changes in the *L. parasitica* DR array may suggest a selective structural feature at the RNA level, but no function has currently been assigned.

**Figure 6:**
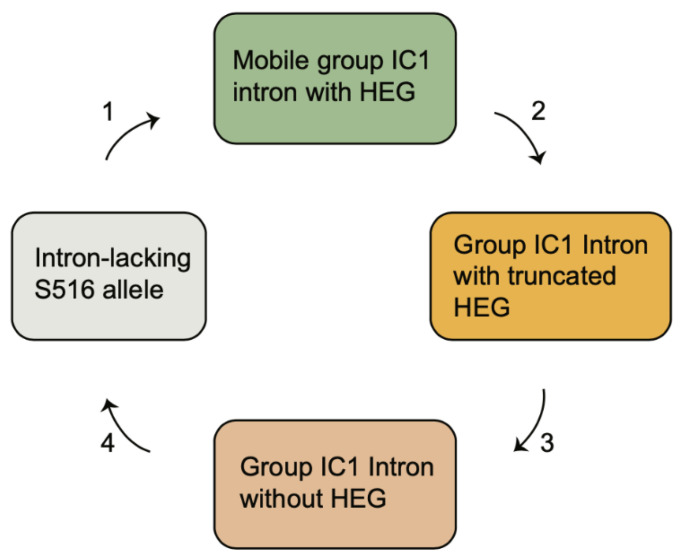
The evolutionary history of nucleolar S516 group I introns based on invasion and extinction.

The S516 group IC1 intron in myxomycetes and other eukaryotic microorganisms constitute a phylogenetic distinct family with a common origin [6,56]. While introns within a short-term evolutionary time frame show strict vertical inheritance patterns [38], most introns indicate a complex evolutionary history not consistent with stable vertical transfers. Based on the idea of the Goddard–Burt cyclic model [57], we propose a scenario for the S516 group I introns that is funded on intron invasion, periodicity of HEG function, and intron loss. The scenario (Figure 6) is described by the four following stages. (1) A HEG-containing intron was gained at site S516 and effectively became spread in a population by homing mobility. Examples of mobile-type introns in myxomycetes are from *Lamproderma*, *Echinostelium*, and *Trichia*, but also from *Naegleria* [38], *Allovhalkampfia* [39], and *Porphyra* [49]. (2) To inactivate homing endonuclease activity, and subsequent intron homing, frameshift, truncations, and sporadic deletions occurred in the HEG region. Such variants have been noted in the S516 introns of *Naegleria martinezi* [38], *Bangia fuscopurpurea* [58], and an *Acanthamoeba* sp. Isolate [49]. (3) The HEG insertion then became completely lost, resulting in an all-ribozyme organization as seen in *D. alpinum*, as well as 93% of the myxomycete S516 introns. HEG deletion was further supported in one of the *Trichia* S516 introns and suggests a link between deletion of HEG and generation of DR. (4) The complete intron became lost, probably due to homologous recombination with an intron-less allele resulting in SSU rDNA lacking an S516 intron, as noted in the approximately 80% of myxomycetes assessed.

## 5. Conclusions

The myxomycete protists represent a unique model system for studying the evolutionary history of self-splicing introns due to an exceptionally high content of nucleolar group I introns. We analyzed 75 group I introns at position 516 in the SSU rDNA of myxomycetes representing five distantly related taxonomic orders. The result reveals a conserved group IC1 ribozyme core, but highly variable peripheral RNA domains containing direct repeat arrays and homing endonuclease genes in 20% of the taxa. All HEGs were interrupted by small spliceosomal introns, which probably facilitate the expression of protein-coding genes embedded in nuclear rDNA. Phylogenetic analysis identified a complex inheritance pattern that can be explained by repeatedly intron gain and loss during the evolution. The current study expands our understanding of the structural organization of group IC1 introns and their evolution.

## Data Availability

New sequencing data are available in GenBank under Accession Number ON155994 (*Diderma alpinum* Fr-K12).

## Supplementary Materials

The following supporting information can be downloaded at: https://www.mdpi.com/article/10.3390/genes13060944/s1, Figure S1: Sequence alignment of 143 core structure nucleotides of myxomycete S516 group I intron. Secondary structure paired segments (P1–P8) are specified with colors and their location is marked above the alignment. Intron sequences are indicated by GenBank accession numbers and family names. Note that DQ903678 is denoted as a *Diderma* sp. (*). Figure S2: Molecular phylogeny of myxomycete taxa-based SSU rDNA sequences. Molecular phylogeny of myxomycete taxa-based SSU rDNA sequences. The SSU-based topology is obtained by neighbor-joining (NJ) analysis of 67 taxa and 1524-nt aligned positions (SSU rDNA dataset 1). Some isolates are left out from the analysis due to partial SSU rDNA sequences. The predicted location of *Reticularia* and *Licea* isolates is shown in gray color letters. The tree is rooted with the *T. varia* SSU rDNA sequence. The NJ, maximum likelihood (ML) and maximum parsimony (MP) bootstrap replicates (≥50%) are given at each node. Black dots at branch points; maximum support in ML, NJ, and ME (≥97%). ^1^ *Tricia varia* clade is composed of twenty *T. varia* isolates (KM495021-27, KM495029, KM495011-13, KM494999, KM495017, KM495005-9, KM495030, and KM495031). Homing endonuclease genes (HEGs) and direct repeats (DRs) are indicated in red and blue letters. The scale bar indicates the fraction of substitutions per site. Figure S3: Molecular phylogeny of myxomycete S516 group I introns. The intron topology is obtained by neighbor-joining (NJ) analysis of 75 taxa and 143-nt aligned positions (intron dataset 2; Figure S1). The NJ and minimal evolution (ME) bootstrap replicates (≥50%) are given for each node. Bold lines indicate maximum support in NJ and ME (≥98%). ^1^ *Tricia varia* clade is composed of twenty *T. varia* isolates (KM495021-27, KM495029, KM495011-13, KM494999, KM495017, KM495005-9, KM495030, and KM495031). Homing endonuclease genes (HEGs) and direct repeats (DRs) are indicated in red and blue letters. The scale bar indicates the fraction of substitutions per site. Figure S4: Two closely related *Meriderma* species contain distantly related S516 group I introns. (a) Secondary structure diagram of *M. aggregarum* S516 intron RNA. (b) Secondary structure diagram of *M. cribrariodes* S516 intron RNA. Peripheral regions with significant structural differences are boxed. Figure S5: (a) Structural features of homing endonuclease genes (HEG)-containing S516 group I intron in *Lamproderma pseudomaculatum*. (b) Nucleotide sequence of HEG, including the 58-nt spliceosomal intron (red letters). Start (ATG) and stop (TAA) codons are indicated, as well as the polyadenylation signal (AATAAA). (c) Amino acid sequence of the homing endonuclease. Important residues involved in Zn binding (red) and catalysis (blue) are indicated. Figure S6: (a) Structural features of homing endonuclease genes (HEG)-containing S516 group I intron in *Echinostelium coelocephalum*. (b) Nucleotide sequence of HEG, including the 59-nt spliceosomal intron (red letters). Start (ATG) and stop (TAA) codons are indicated, as well as the polyadenylation signal (AATAAA). (c) Amino acid sequence of the homing endonuclease. Important residues involved in Zn binding (red) and catalysis (blue) are indicated. Figure S7: (a) Structural features of homing endonuclease genes (HEG)-containing S516 group I intron in *Trichia varia*. (KM494993-5). (b) Nucleotide sequence of HEG, including the 50-nt spliceosomal intron (red letters). Start (ATG) and stop (TGA) codons are indicated, as well as the polyadenylation signal (AATAAA). (c) Amino acid sequence of the homing endonuclease. Important residues involved in Zn binding (red) and catalysis (blue) are indicated. Figure S8: Sequence alignment of direct repeat motifs in myxomycete S516 group I intron. Direct repeats are located in peripheral regions P1, P2, P5, P6, P8, and P9. Figure S9: RNA structural support of HEG deletion in *Trichia varia* S516 group I introns. HEG deletion in segment P2 (KM494996) leaves a HEG remnant sequence corresponding to the 5′ end of the open reading frame (indicated by green colored nucleotides), as well as a direct repeat feature (DR-1 and DR-2). Only paired segments P1 and P2 are shown. Table S1: Key features of 75 nucleolar group I introns at position S516 in myxomycete. Table S2: Schematic organization of direct repeats arrays in S516 group I introns in myxomycetes.
