# Supplementary material for: Structural Organization of S516 Group I Introns in Myxomycetes
Source: Genes (Basel). 2022 May 25;13(6):944. doi: 10.3390/genes13060944 (PMC9223047; doi:10.3390/genes13060944)
Supplement: Supplementary file 1 [file genes-13-00944-s001.zip › genes-1737068-supplementary.pdf]

Figure S1. Sequence alignment of core structure nucleotides of myxomycete S516 introns

INTRON

```
                              <IGS>    <<<P3>>>    <<P4>>    <<P5> <<<P5a>>>>
Dalp.S516_ON155994      CTGGTTAGAGCCAGGTACAGATGTAAATTGCAGGAAACTCTGGAGTCATAGCTACCGGTAATAACGCTAT
Dniv.S516-1_HE614615    CTGGTGGACGCCAGGTACAGATGTAAATTGCAGGGACGTCTGAAGTCACGAATACCGGTAATAACGTCGT
Dniv.S516-2_HE614616    CTGGTGGACGCCAGGTACAGATGTAAATTGCAGGGACGTCTGAAGTCACGAATACCGGTAATAACGTCGT
Dniv.S516-3_HE614617    CTGGTGGACGCCAGGTACAGATGTAAATTGCAGGGACGTCTGAAGTCACGAATACCGGTAATAACGTCGT
Dsou.S516_MZ313549      CTGGTTGTAGCCAGGTACGGACGTAAACTGCAGGAAACTCTGGAGCCTAGGCTACCGGTAACAACGCCTA
Lcru.S516_HE614619      CTGGTTGAAGCCAGGTACAGCCGTAAGTTGCGGGGAACGCCAGTGGGGGGGATACCGGTAACAAAGCCCC
Dsp.S516*_DQ903678      CTGGTGGAAGCCAGGTACAGATGTAAACTGCAGGGACGTCTGGAGTCATGACTACCGGTAATAACGTTAT
Bgra.S516_AJ555451      CTGGTTATAGCCAGGTACAGTTGTAAATTGCGGGGAACACCAGTGATTGGACTACCGGTAATAACGTCCA
Flev.S516_DQ903676      CTGGTTACAGCCAGGTACAGCCGTAAATTGCGAGGAACACCAGTGACTGGGCTACCGGTAACAATGCTCA
Fsep.S516-1_AJ584697    CTGGTAAATGCCAGGTACAGCTGTAAATTGCGGGGAACGCCAGTGGGAAAGCTACCGGTAACAATGCGAT
Fsep.S516-2_MF983561    CTGGTGTATGCCAGGTACAGCTGTAAATTGCGGGGAACGCCAGTGGGAAAGCTACCGGTAACAATGCGAT
Fsep.S516-3_AJ555452    CTGGTAAATGCCAGGTACAGCTGTAAATTGCGGGGAACGCCAGTGGGAAAGCTACCGGTAACAATGCGAT
Ecer.S516_JQ031967      CTGGTTTTAGCCAGGTACACTCGTAAATTGCGGGAAACACTGGGGAGTCAGCGACATGTAATACTGTTGA
Pfla.S516_AJ555453/4    CTGGTTGAAGCCAGGTACACGCGTAAACTGCGGGGAACACTGGGGATTCATGTACCGGTAAAACCATGGA
Pnot.S516_JX035988      CTGGTGGATGCCAGGTACATTCGTAAATTGCGGGGAAGGCTGGTGGGAGTGCCACGCGTAACAGCGCACT
Psp.S516-1_JX035984     CTGGTGGCAGCCAGGTACAGTTGTAAATTGCGGGGAACACCAGTGACTGGACTACCGGTAACAACGTCCA
Psp.S516-2_MK336180     CTGGTTGTAGCCAGGTACGACTGTAAATTGCAGGGAACGCTAGGGGCGTAGATACCGGTGATAATGCTAC
Cana.S516_JQ031961      CTGGTTGAAGCCGGTTACAGCTTTAAACTGCAAGGAAAACTGGTGAAGCGGATACGCGTTATAACGCCGC
Cnig.S516-1_DQ903685    CTGGTGGACGCCAGGTACAGACGTAAATTGCAGGGAACTCTGGGTGATCGACTACCGGTAATAACGTCGG
Cnig.S516-2_AY643824    CTGGTGGACGCCAGGTACACTTGTAAATTGCGGGGAACGCTGATGAGGTGGGTACCGGTAAAAACCCCGC
Cpse.S516_DQ903673      CTGGTTGTTGCCAGATACAGCTGTAAACTGCAGGGAAAACTGGTGGAGCGGATACGCGTTATAACGCTGC
Cocu.S516_JQ031959      CTGGTTAAAGCCAGGTACACGTGTAAATTGCAGGGAACGCCTAGGGATAGACCACCGGTAACAGCGTCTA
Crob.S516_JQ031960      CTGGTTAACGCCAGGTACACCTGTAAATTGCGGGGAACACTGGGGATCAAGCTACGCGTAATAACGCTTG
Dpau.S516_JQ031966      CTGGTTAGAGCCAGGTACAGTTGTAAATTGCGGGGAAAGTCAGGGATCACAGCACCGGTAAAAGCCTGTG
Laca.S516_JQ031968      CTGGTTAAAGCCAGGTACAGTTGTAAATTGCGGGGAAGGCCAGTGAGTGCAGCACCGGTAATAGCCTGCA
Laen.S516-1_JQ031969    CTGGTTACAGCCAGGTACAGTTGTAAATTGCGGGGAACACCAGTGGGCACAGCACCGGTAATAGTCTGTG
Laen.S516-2_JQ031970    CTGGTTACAGCCAGGTACAGTTGTAAATTGCGGGGAACACCAGTGGGCACAGCACCGGTAATAGTCTGTG
Lalb.S516_JQ031971      CTGGTTGGAGCCAGGTACAGTTGTAAATTGCGGGGAACACCAGTGGGCACAGCACCGGTAACAGTCTGTG
Lcol.S516-1_HQ687204    CTGGTTACAGCCAGTTACAGTTGTAAATTGCGGGGAAGACCAGTGGGAACAGCACCGGTAATAGCCTGTC
Lcol.S516-2_HQ687196    CTGGTCCGGGCCAGGTACACTTGTAAATTGCAGGGAAAGCCTAAGAAAATGCCACCGGTAAAAGCGCTCT
Lcol.S516-3_HQ687198    CTGGTGAACGCCAGGTACATTTGTAAATTGCAGGGAACACTGGAGGGTCAGCTACGCGTAATAACGCTGA
Lcol.S516-4_HQ687199    CTGGTACTTGCTGGTTACATCCGTAAATTGCAGGAAAAGCCTTAAGGAAGGAGCCCGGTAACAGCGCTCT
Lcol.S516-5_HQ687197    CTGGTTATAGCCAGGTACCCTTGTAAATTGCGGGAAACCCTGGCCAACTTCGGGCGCGTAATAATGCTGG
Lcol.S516-6_HQ687200    CTGGTTACAGCCAGTCACAGTTGTAAATTGCGGGGAACACCAGTGGGAACAGCACCGGTAATAGCCTGTC
Lech.S516-1_JQ031980    CTGGTGAGCGCCAGGTACAGATGTAAATTGCAGGGAAGTCTGGGTGAACGGATACCGGTAAGAACTCCTT
Lech.S516-2_JQ031979    CTGGTGAGCGCCAGGTACAGATGTAAATTGCAGGGAAGTCTGGAGTGATGGATACCGGTAAGAACTCCGT
Lpse.S516_JQ031985      CTGGTTGGAGCCAGGTACAATTGTAAAATTCGGGGAAAACCCGTGGGAACAACCCCGGTAAAAACCTTTT
Lpun.S516_HQ687194      CTGGTGTACGCCAGGTACACTCGTAAATTGCGGGGAAAACCTGAGGAAGGACCACCGGTAACAGCGTCTT
Lsp.S516_JQ031994       CTGGTTACAGCCAGGTACAGTTGTAAATTGCAGGAAAAGCCAGTGGGTGCAGCACCGGTAATAGCCTGCA
Mobl.S516_DQ903682      CTGGTAGTTGCCAGGTACACTTGTAAATTGCGGGAAACACTGGATGTGTGCGTACGCGTACATCGCACAC
Magg.S516_DQ903669      CTGGTTGGAGCCAGGTACAGTTGTAAATTGCGGGGAACACCAGAGGTCATAGTACCGGTAATAATCTATG
Mcri.S516_JQ032000      CTGGTGTTCGCCAGGTACAGTTGTAAACTGCGGGAAAGGCTTGGGGGGTTGGTACCGGTAATAACCCAAC
Sfus.S516-1_MK041079    CTGGTCAACGCCAGGTACAACTGTAAATTGCAGGGAACACCAGGGGTGGAAGTACCGGTTACAATCTTCC
Sfus.S516-2_MK041078    CTGGTGGACGCCAGGTACAACTGTAAATTGCAGGGAACACCAGTGGGTGGGGTACCGGTAACAATCCTCA
Lpar.S516_JX481297      CTGGTGGACGCCAGGTACATGTGTAAACTGCGAGGACGTCCAGAGGGATAGCTACCGGTAATCAACGCTA
Rlyc.S516_JX481311      CTGGTTGCAGCCAGGTACGCTCGTAAATTGCGGGAAGGATGGGGTGTGGGCCTACGCGTAAAAACGGCCT
Aglo.S516_JX481282      CTGGTTACAGCCAGGTACAGTTGTAAATTGCAGGGAACACCAGTGGTTCAGCTACCGGTAACAACGCTGT
Tdec.S516_FJ810499      CTGGTTATAGCCAGGTACAATCGTAAATTGCAGGGAACGCTAGGGGTATGGATACTGGTGAAAACGCCAT
Tvar.S516-1_KM495030    CTGGTTGTAGCCAGGTACAGCTGTAAATTGCAGGGAACACTAGGGGTGTGGATACCGGTGACAACGCCAC
Tvar.S516-2_KM495011    CTGGTTGTAGCCAGGTACAGCTGTAAATTGCAGGGAACACTAGGGGTGTGGATACCGGTGACAACGCCAC
Tvar.S516-3_KM495025    CTGGTTGTAGCCAGGTACAGCTGTAAATTGCAGGGAACACTAGGGGTGTGGATACCGGTGACAACGCCAC
Tvar.S516-4_KM495027    CTGGTTGTAGCCAGGTACAGCTGTAAATTGCAGGGAACACTAGGGGTGTGGATACCGGTGACAACGCCAC
Tvar.S516-5_KM495026    CTGGTTGTAGCCAGGTACAGCTGTAAATTGCAGGGAACACTAGGGGTGTGGATACCGGTGACAACGCCAC
Tvar.S516-6_KM495022    CTGGTTGTAGCCAGGTACAGCTGTAAATTGCAGGGAACACTAGGGGTGTGGATACCGGTGACAACGCCAC
Tvar.S516-7_KM495010    CTGGTTGTAGCCAGGTACAGCTGTAAATTGCAGGGAACACTAGGGGTGTGGATACCGGTGACAACGCCAC
Tvar.S516-8_KM495009    CTGGTTGTAGCCAGGTACAGCTGTAAATTGCAGGGAACACTAGGGGTGTGGATACCGGTGACAACGCCAC
Tvar.S516-9_KM494999    CTGGTTGTAGCCAGGTACAGCTGTAAATTGCAGGGAACACTAGGGGTGTGGATACCGGTGACAACGCCAC
Tvar.S516-10_KM495017   CTGGTTGTAGCCAGGTACAGCTGTAAATTGCAGGGAACACTAGGGGTGTGGATACCGGTGACAACGCCAC
Tvar.S516-11_KM495008   CTGGTTGTAGCCAGGTACAGCTGTAAATTGCAGGGAACACTAGGGGTGTGGATACCGGTGACAACGCCAC
Tvar.S516-12_KM494996   CTGGTTGCAGCCAGGTACGACCGTAAATTGCAGGGAACGCCAGGGGAGCAGATACCGGTGATAACGCTGC
Tvar.S516-13_KM495029   CTGGTTGTAGCCAGGTACAGCTGTAAATTGCAGGGAACACTAGGGGTGTGGATACCGGTGACAACGCCAC
Tvar.S516-14_KM495023   CTGGTTGTAGCCAGGTACAGCTGTAAATTGCAGGGAACACTAGGGGTGTGGATACCGGTGACAACGCCAC
Tvar.S516-15_KM495012   CTGGTTGTAGCCAGGTACAGCTGTAAATTGCAGGGAACACTAGGGGTGTGGATACCGGTGACAACGCCAC
Tvar.S516-16_KM495024   CTGGTTGTAGCCAGGTACAGCTGTAAATTGCAGGGAACACTAGGGGTGTGGATACCGGTGACAACGCCAC
Tvar.S516-17_KM495021   CTGGTTGTAGCCAGGTACAGCTGTAAATTGCAGGGAACACTAGGGGTGTGGATACCGGTGACAACGCCAC
Tvar.S516-18_KM494993   CTGGTGGTAGCCAGGTACAACCGTAAATTGCAGGGAACGCCAGGGGAGCAGATACCGGTGATAACGCTGC
Tvar.S516-19_KM495013   CTGGTTGTAGCCAGGTACAGCTGTAAATTGCAGGGAACACTAGGGGTGTGGATACCGGTGACAACGCCAC
Tvar.S516-20_KM495007   CTGGTTGTAGCCAGGTACAGCTGTAAATTGCAGGGAACACTAGGGGTGTGGATACCGGTGACAACGCCAC
Tvar.S516-21_KM495006   CTGGTTGTAGCCAGGTACAGCTGTAAATTGCAGGGAACACTAGGGGTGTGGATACCGGTGACAACGCCAC
Tvar.S516-22_KM495005   CTGGTTGTAGCCAGGTACAGCTGTAAATTGCAGGGAACACTAGGGGTGTGGATACCGGTGACAACGCCAC
Tvar.S516-23_KM495031   CTGGTTGTAGCCAGGTACAGCTGTAAATTGCAGGGAACACTAGGGGTGTGGATACCGGTGACAACGCCAC
Tvar.S516-24_KM494995   CTGGTGGTAGCCAGGTACAACCGTAAATTGCAGGGAACGCCAGGGGAGCAGATACCGGTGATAACGCTGC
Tvar.S516-25_KM494994   CTGGTGGTAGCCAGGTACAACCGTAAATTGCAGGGAACGCCAGGGGAGCAGATACCGGTGATAACGCTGC
Dniv.S516_JX481289      CTGGTCACAGCCAGCTGCAACGGGAAATTAACCGGAAGCAGCCGGGGTCCGATGATTCAGGGCCTGTCCG
Ecoe.S516_AY842033      CTGGTTACAGCCAGGTACAATTGTAAATTGCGGGAAACACCAGTGGAGAGACCACCGGTAAACAGCGTCT
```

INTRON

```
                  P5a> <P5>>   <<P4>><<P6>>    <<P7>>>    <<P3>>><<P8>>        <<P7>>
Dalp.S516         GGCACCAGACAATCGGCAGCCGGTTCAGAGACTATAATCGTCTGGGCGCTTAAGGAATAGTCCATTAGGCCAG
Dniv.S516-1       GGCACCAGATAATCGGCAGCCGGTTCAGAGACTATAATCATCTGGGCGCTTAAGGAATAGTCCATTAGGCCAG
Dniv.S516-2       GGCACCAGATAATCGGCAGCCGGTTCAGAGACTATAATCATCTGGGCGCTTAAGGAATAGTCCATTAGGCCAG
Dniv.S516-3       GGCACCAGATAATCGGCAGCCGGTTCAGAGACTATAATCATCTGGGCGCTTAAGGAATAGTCCATTAGGCCAG
Dsou.S516         TGGCCCAGACAATCGGCAGCCGGTTCAGAGACTATAATCGTCCGGGCGCTCAAGGAATAGTCCATTAGGCCAG
Lcru.S516         TTCACTGGTCAATCCGCAGCCGGTTCAGAGACTATAATCGGTTGGGCGCCTAAGGGATAGTCCACTGGGCCAG
Dsp.S516*         GGCATCAGATAATCGGCAGCCGGTTCAGAGACTATAATCATCTGGGCGCTTAAGGAATAGTCCATTAGGCCAG
Bgra.S516         CTCACTGGTCAATCCGCAGCCGGTTCAGAGACTATAATCGACTGGGCGCTTAAGGAATAGTCCACTGGGCCAG
Flev.S516         CTCACTGGTCAATTCGCAGCCGGTTCAGAGACTATAATCGGTTGGGCGCTTAAGGAATAGTCCACTGGGCCAG
Fsep.S516-1       CCAGCTGGCCAATCCGCAGCCGGTTCAGAGACTATAATCAGTTGGGCGCTTAAGGGATAGTCCACTGGGCCAG
Fsep.S516-2       CCCACTGGCCAATCCGCAGCCGGTTCAGAGACTATAATCAGTTGGGCGCTTAAGGGATAGTCCACTGGGCCAG
Fsep.S516-3       CCAGCTGGCCAATCCGCAGCCGGTTCAGAGACTATAATCAGTTGGGCGCTTAAGGGATAGTCCACTGGGCCAG
Ecer.S516         TTCCCCAGTCAATCCGCAGCCGGTTCAGAGACTATAATCGAGTGGGGTCTTAATGTATAGTCCACTGGGCCAG
Pfla.S516         TTCGTCAGTCAATCCGCAGCCGGTTCAGAGACTATAATCGTGTGGGGTCTTAAGGTATAGTCCACTGGGCCAG
Pnot.S516         CTCACCAGTCAATCCGCAGCCGGTTCAAAGACTACAATCGAATGGGGCCTTAAGGTATAGTCCACTGGGCCAG
Psp.S516-1        CTCACTGGTCAATCCGCAGCCGGTTCAGAGACTATAATCAACTGGGCGCTTAAGGAATAGTCCACTGGGCCAG
Psp.S516-2        GCTCCTAGCCAATCTGCAGCCGGTTCAGAGACTATAATCAGTTGGGCGCTTAAGGAATAGTCCATTAGGCCAG
Cana.S516         TTCACCAGTTTATTTGCCGCCGGTTCAGAGACTACAATCAGCTGGGGCTTTAAGGAATAGTCCACTGGGCCAG
Cnig.S516-1       TTGCCCAGACAATCTGCAGCCGGTTCAGAGACTATAATCGTTTGGGCGCTTAAGGAATAGTCCACTAGGCCAG
Cnig.S516-2       TTCATTAGCCAATCCGCAGCCGGTTCAGAGACTATAATCAGGTGGGGTCTTAAGGTATAGTCCACTAGGCCAG
Cpse.S516         TTCACCAGTCTATCTGCCGCCGGTTCAGAGACTACAATCAGCTGGGGCTTTAAGGAATAGTCCACTGGGCCAG
Cocu.S516         TTCCCAGGCCAATCCGCAGCCGGTTCAGAGACTATAATCATGTGGGGCCTTAAGGGATAGTCCACTGGGCCAG
Crob.S516         GTCCTCAGTCAATCCGCAGCCGGTTCAGAGACTATAATCAGGTGGGGCCTTAAGGTATAGTCCACTGGGCCAG
Dpau.S516         TCTTCTGATCGATCCGCAGCCGGTTCAGAGACTACAATCGACTGGGGCCTTAAGGTATAGTCCACTGGGCCAG
Laca.S516         ACTCCTGGGCAATCCGCAGCCGGTTCAGAGACTACAATCGACTGGGGTATTAAGGTATAGTCCACTGGGCCAG
Laen.S516-1       CCTTCTGGTCAATCCGCAGCCGGTTCAGAGACTATAATCGACTGGGGTCTTAAGGTATAGTCCACTGGGCCAG
Laen.S516-2       CCTTCTGGTCAATCCGCAGCCGGTTCAGAGACTATAATCGACTGGGGTCTTAAGGTATAGTCCACTGGGCCAG
Lalb.S516         CCTTCTGGTCAATCCGCAGCCGGTTCAGAGACTATAATCGACTGGGGTCTTAAGGTATAGTCCACTGGGCCAG
Lcol.S516-1       TCCTCTGGTTAATCTGCAGCCGGTTCAGAGACTATAATCGACTGGGGCCTTAAGGCATAGTCCACTGGGCCAG
Lcol.S516-2       TCCGCAGGCCAATCCGCAGCCGGTTCAGAGACTATAATCGAGTGGGGCCTTAAGGGATAGTCCACTGGGCCAG
Lcol.S516-3       CTCCCCAGTCAATCCGCAGCCGGTTCAGAGACTATAATCGAGTGGGGCTCCTAAGGATAGTCCACTGGGCCAG
Lcol.S516-4       TTCCGAGGCCAACCCGCAGCCGGTTCAGAGACTATAATCGGGTGGGGCCTTAATGGATAGTCCACTGGGCCAG
Lcol.S516-5       CTCCCCAGTCAATCCGCAGCCGGTTCAGAGACTATAATCAAGTGGGGTCTTAAGGGATAGTCCACTGGGCCAG
Lcol.S516-6       TCCTCTGGTCAATCTGCAGCCGGTTCAGAGACTATAATCAACTGGGGCCTTAACGAATAGTCCACTGGGCCAG
Lech.S516-1       CACATCAGATAATCTGCAGCCGGTTCAGAGACTATAATCATCTGGGCGCCTAAGGAATAGTCCAATTGGCCAG
Lech.S516-2       CACATCAGACAATCTGCAGCCGGTTCAGAGACTATAATCATCTGGGCGCCTAAGGAATAGTCCAATTGGCCAG
Lpse.S516         TCCCCTGGTCAATTCGCAACCGGTTCAGAGACTACAATCGACTGGGGTCTTAAGGTATAGTCCACTGGGCCAG
Lpun.S516         TCCTCAGGCCAATCCGCAGCCGGTTCAGAGACTATAATCGAGTGGGGTCTTAATGGATAGTCCACTGGGCCAG
Lsp.S516          CCTTCTGGGTAATCTGCAGCCGGTTCAGAGACTATAATCAACTGGGGTCTTAAGGTATAGTCCACTGGGCCAG
Mobl.S516         CGGACCAGTCAATCCGCAGCCGGTTCAGAGACTATAATCAGGTGGGCGCTTAAGGGATAGTCCATTAGGCCAG
Magg.S516         GCTGCTGGTCAATCCGCAGCCGGTTCAGAGACTACAATCGACTGGGGCCTTAAGGTATAGTCCACTAGGCCAG
Mcri.S516         CCTCCAAGGCAACCTGCAGCCGGTTCAGAGACTACAATCGACTGGGGCCTTAAAGCATAGTCCAATTGGCCAG
Sfus.S516-1       ATTCCTGGTCAATCTGCAGCCGGTTCAGAGACTATAATCAGTTGGGCGCTTAAGGAATAGTCCACTGGGCCAG
Sfus.S516-2       TTCCCTGGTCAATCTGCAGCCGGTTCAGAGACTATAATCAGTTGGGCGCTTAAGGAATAGTCCACTGGGCCAG
Lpar.S516         TCACTTGGATAATTTGCAGCGCGTTCAGAGACTATAATCACGTGGGGTCTTAAGGCATAGTCCACTGGGCCAG
Rlyc.S516         CACACCATTTAATCCGCAGCCGGTCCAGAGACTATACACGAGCGGGGCCCTAAGATATAGTCCACTGGGCCAG
Aglo.S516         TCCACTGGTCAATCTGCAGCCGGTTCAGAGACTATAATCGACTGGGCGCTTAAGGAATAGTCCACTGGGCCAG
Tdec.S516         GCTCCTAGCCAATCTGCAGCCGGTTCAGAGACTATAATCGGTTGGGCGCTTAAGGAATAGTCCACTGGGCCAG
Tvar.S516-1       ACTCCTAGTCAATCTGCAGCCGGTTCAGAGACTATAATCAGCTGGGCGCTTAAGGAATAGTCCACTGGGCCAG
Tvar.S516-2       ACTCCTAGTCAATCTGCAGCCGGTTCAGAGACTATAATCAGCTGGGCGCTTAAGGAATAGTCCACTGGGCCAG
Tvar.S516-3       ACTCCTAGTCAATCTGCAGCCGGTTCAGAGACTATAATCAGCTGGGCGCTTAAGGAATAGTCCACTGGGCCAG
Tvar.S516-4       ACTCCTAGTCAATCTGCAGCCGGTTCAGAGACTATAATCAGCTGGGCGCTTAAGGAATAGTCCACTGGGCCAG
Tvar.S516-5       ACTCCTAGTCAATCTGCAGCCGGTTCAGAGACTATAATCAGCTGGGCGCTTAAGGAATAGTCCACTGGGCCAG
Tvar.S516-6       ACTCCTAGTCAATCTGCAGCCGGTTCAGAGACTATAATCAGCTGGGCGCTTAAGGAATAGTCCACTGGGCCAG
Tvar.S516-7       ACTCCTAGTCAATCTGCAGCCGGTTCAGAGACTATAATCAGCTGGGCGCTTAAGGAATAGTCCACTGGGCCAG
Tvar.S516-8       ACTCCTAGTCAATCTGCAGCCGGTTCAGAGACTATAATCAGCTGGGCGCTTAAGGAATAGTCCACTGGGCCAG
Tvar.S516-9       ACTCCTAGTCAATCTGCAGCCGGTTCAGAGACTATAATCAGCTGGGCGCTTAAGGAATAGTCCACTGGGCCAG
Tvar.S516-10      ACTCCTAGTCAATCTGCAGCCGGTTCAGAGACTATAATCAGCTGGGCGCTTAAGGAATAGTCCACTGGGCCAG
Tvar.S516-11      ACTCCTAGTCAATCTGCAGCCGGTTCAGAGACTATAATCAGCTGGGCGCTTAAGGAATAGTCCACTGGGCCAG
Tvar.S516-12      TTTCCTGGTCAATCTGCAGCCGGTTCAGAGACTATAATCGGTTGGGCGCCTAAGGAATAGTCCACTGGGCCAG
Tvar.S516-13      ACTCCTAGTCAATCTGCAGCCGGTTCAGAGACTATAATCAGCTGGGCGCTTAAGGAATAGTCCACTGGGCCAG
Tvar.S516-14      ACTCCTAGTCAATCTGCAGCCGGTTCAGAGACTATAATCAGCTGGGCGCTTAAGGAATAGTCCACTGGGCCAG
Tvar.S516-15      ACTCCTAGTCAATCTGCAGCCGGTTCAGAGACTATAATCAGCTGGGCGCTTAAGGAATAGTCCACTGGGCCAG
Tvar.S516-16      ACTCCTAGTCAATCTGCAGCCGGTTCAGAGACTATAATCAGCTGGGCGCTTAAGGAATAGTCCACTGGGCCAG
Tvar.S516-17      ACTCCTAGTCAATCTGCAGCCGGTTCAGAGACTATAATCAGCTGGGCGCTTAAGGAATAGTCCACTGGGCCAG
Tvar.S516-18      TTTCCTGGTCAATCTGCAGCCGGTTCAGAGACTATAATCGGTTGGGCGCCTAAGGAATAGTCCACTGGGCCAG
Tvar.S516-19      ACTCCTAGTCAATCTGCAGCCGGTTCAGAGACTATAATCAGCTGGGCGCTTAAGGAATAGTCCACTGGGCCAG
Tvar.S516-20      ACTCCTAGTCAATCTGCAGCCGGTTCAGAGACTATAATCAGCTGGGCGCTTAAGGAATAGTCCACTGGGCCAG
Tvar.S516-21      ACTCCTAGTCAATCTGCAGCCGGTTCAGAGACTATAATCAGCTGGGCGCTTAAGGAATAGTCCACTGGGCCAG
Tvar.S516-22      ACTCCTAGTCAATCTGCAGCCGGTTCAGAGACTATAATCAGCTGGGCGCTTAAGGAATAGTCCACTGGGCCAG
Tvar.S516-23      ACTCCTAGTCAATCTGCAGCCGGTTCAGAGACTATAATCAGCTGGGCGCTTAAGGAATAGTCCACTGGGCCAG
Tvar.S516-24      TTTCCTGGTCAATCTGCAGCCGGTTCAGAGACTATAATCGGTTGGGCGCCTAAGGAATAGTCCACTGGGCCAG
Tvar.S516-25      TTTCCTGGTCAATCTGCAGCCGGTTCAGAGACTATAATCGGTTGGGCGCCTAAGGAATAGTCCACTGGGCCAG
Dniv.S516         GCCACTAGCCAATCTGCAGCCGGTTCAGAGACTATAATCGACTGGGCGCTTAAAGAATAGTCCACTCCGCCAG
Ecoe.S516         CTTCCTGGTCAATCCGCAGCCGGTTCAGAGACTATAATCAATTGGGCGCTTAAAGTATAGTCCACTGGGCCAG
```

Molecular phylogeny of myxomycete taxa based on SSU rDNA sequences

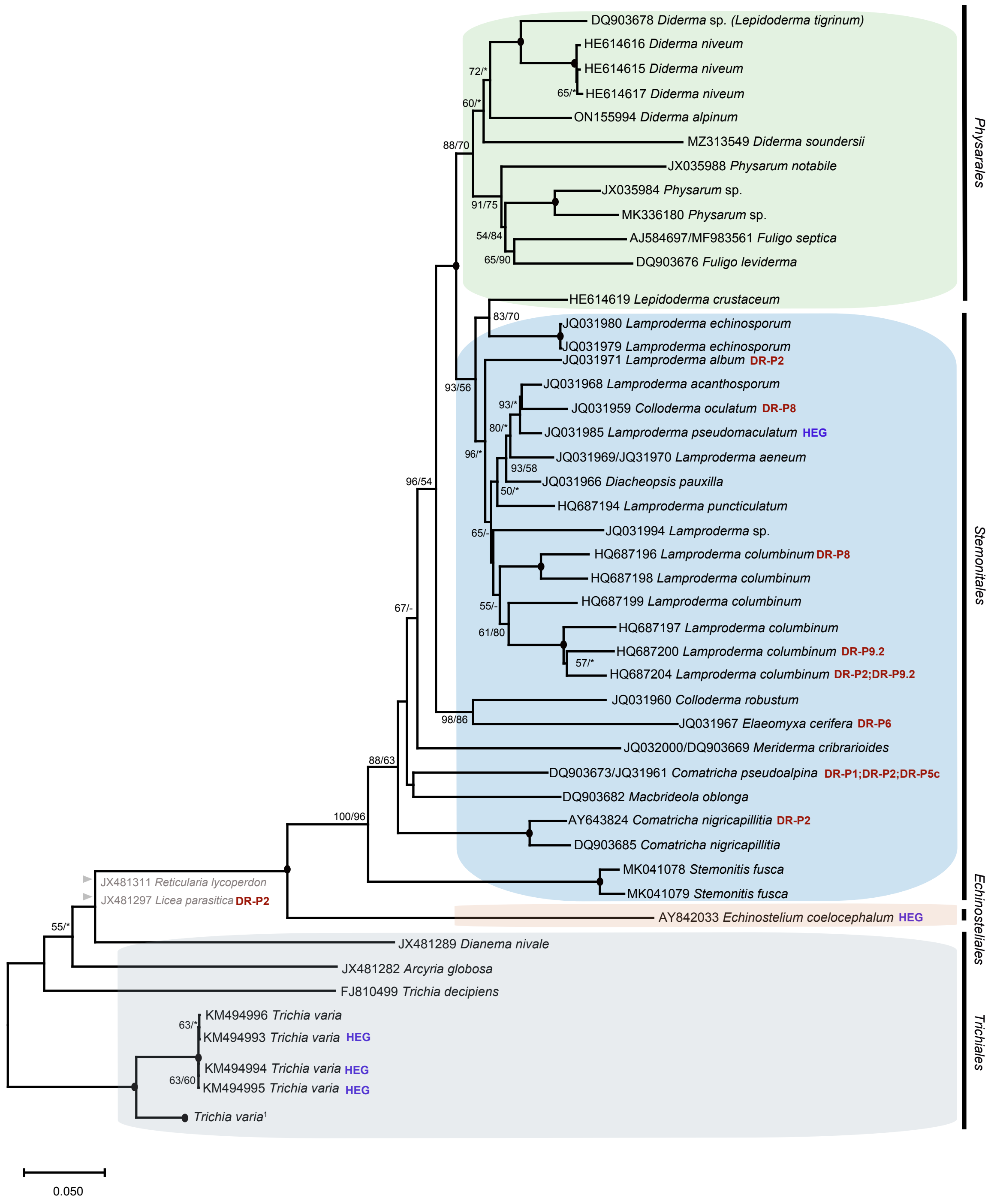

Figure S2

Molecular phylogeny of myxomycete S516 group I introns

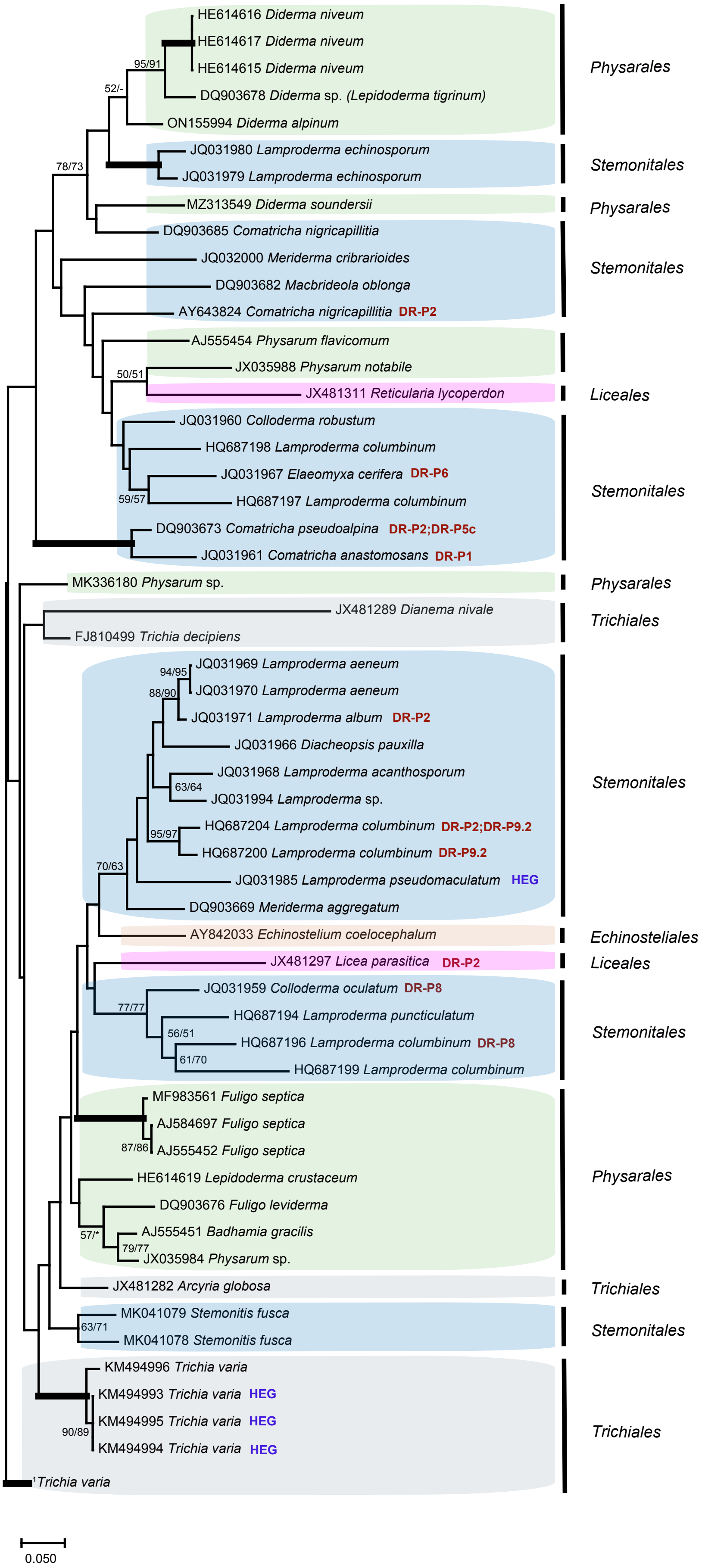

Figure S3

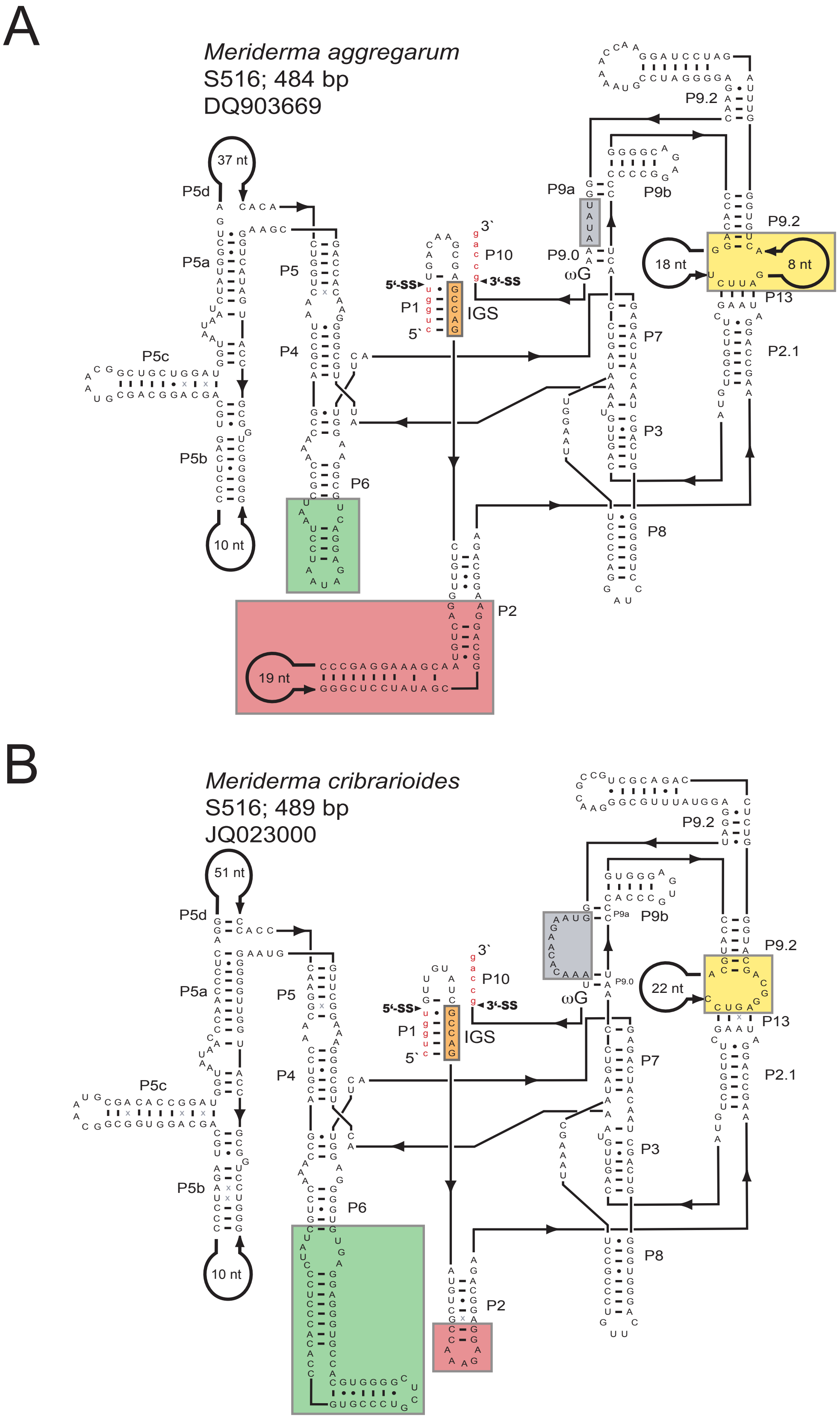
A
Meriderma aggregarum
S516; 484 bp
DQ903669
B
Meriderma cribrarioides
S516; 489 bp
JQ023000
37 nt
51 nt
18 nt
8 nt
22 nt
10 nt
19 nt
P1
P2
P2.1
P3
P4
P5
P5a
P5b
P5c
P5d
P6
P7
P8
P9.0
P9a
P9b
P9.2
P10
P13
IGS
5`
3`
5'-SS
3'-SS
ωG

Figure S4

A

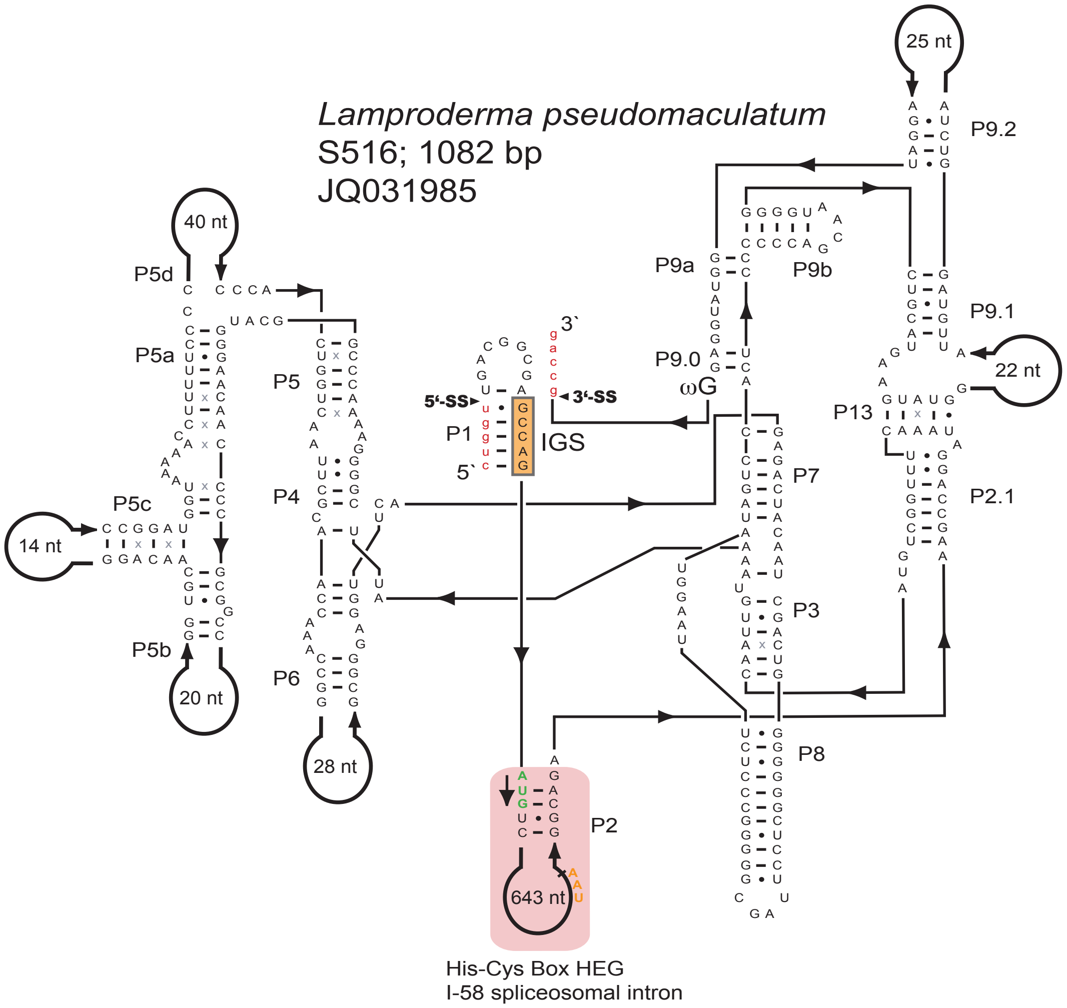

B

ATGTCGAACAATGAGGGAACAACCGAACGTCGAAAGCACAACAAGTCTGGGCTGACCACGCTGAGCGTACTGAAG
TCGAGTGATGAAATGATCCTGGCGCTAGAGCCTCTCCTAAAAAGGAAGGCACGGGCAATAGGCTTCGAAGTAGTC
AAGCGATCCAGGTGCAAAGTATGGCCGAACAAAAAGTCCAAGATCACCAAGGGCAAG**GTATGGCGGGCTGAGCTG**
AACCGAAAGATCGGGCATGAACTAACCACTCGCTGCCCAGGACGTCGGGGAGGCCTATCATGTTAGGGCATTTCA
AGTATTCGGCCGGGAACGTTTGGGGCTGGTACCCCCGAACAAGCGCCAAGGCGACCTCGTGGTATCGCATATATG
CGGGACCCGTCACTGCCTGAAGCGTACCCACCTGCTGCTGGAGCCAAAGTGGGTGAATGACGAACGAACCCACTG
CCACTTTCTGCCACATTCCATGGCGGCGAGGGGCAAGGATCCGGCGGAGACTGTGAAGGCTGTTAGAAAGGCGTG
CCCTCATGAGCCTAGGTGCTTCTCGTACCTGGGCAATTTCGAACCTGATGAACGTGTAATTGTGAGGCCTGTGCC
AGTGGT**AATAAA**CACCATG**TAA**

C

MSMSNNEGTTERRKHNKSGLTTLSVLKSSDEMILALEPLLKRKARAIGFEVVKRSR**C**KVWPNKKSKITKGKDVGE
AYHVRAFQVFGRERLGLVPPNKRQGDLVVS**H**I**C**GTRH**C**LKRT**H**LLLEPKWV**N**DERTH**C**HFLPHSMAARGKDPAET
VKAVRKA**C**P**H**EPR**C**FSYLGNFEPDERVIVRPVPVVINTM   187

A

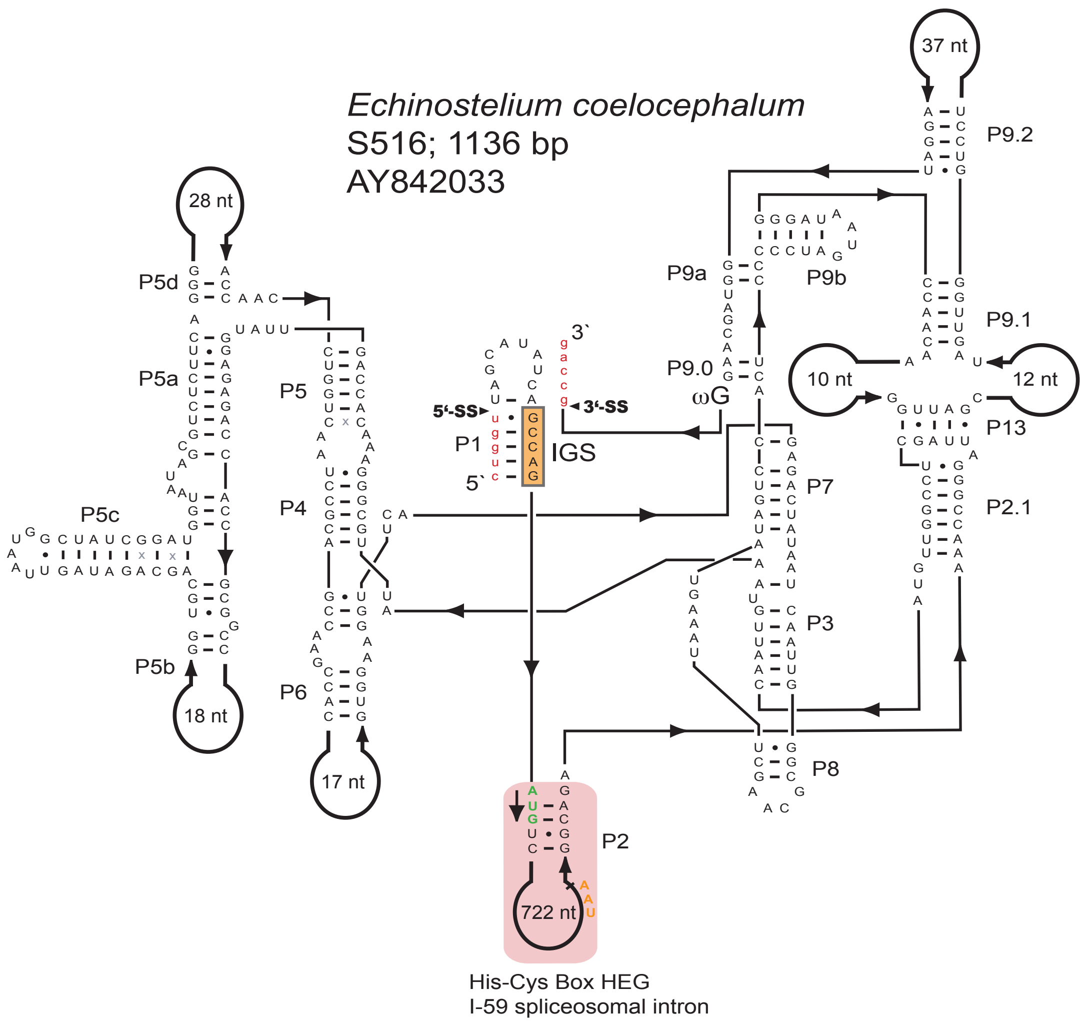

B

ATGTCTGATAGGCTACACAACAAATCGGGTTTAACTGGCTTATCTTTTTTGCAGGAAGATGATACTGTCCTTGCG
GAGTGGACGAAGACTTTGAATAGGCGGGTGAAGGACGGCAAAGAGGCGCGAAGTGCCCCAAAGGTGATTCCACAC
TCTCGCTGTCGGCTACTTGCCAACCACAAAGGCACTTTGGTCATGAGCTCAAAG**GTATGATCATTTTGGGATTGG**
GATTAGGGTTAACTGTATACTGATGGATTTACATACAGCATGTCTGCTACGGCTACCAACTACGTGCTTTGGAGG
TGTTCGGAAGGGATAGGATGGCCCAGGTTCCTCCCAACAAGGCTGGAGACTCCCTTTTGATATCGCATGTCTGTG
GTACTCGGAACTGCTGCCGACGCACGCACCTTGTGATTGAGCCGAAGCACATCAATGATGAGCGGGTGCACTGCC
ACATGGTCGCGCACTGGATGTGGAAGAAGTCAAAGCGCTACGGCAAACGGATGGCAAAGGTTAGGGCGGCAGTCC
AGCTTGCTTGCCCTCACGAGCCAAAGTGTCTTACAAGTGTACGGGATCTGGATGTTAAAGACCGCTTTGTAGAGT
CCAAGCCGAGTAATGC**AATAAA**ATATGAT**TAA**

C

MSDRLHNKSGLTGLSFLQEDDTVLAEWTKTLNRRVKDGKEARSAPKVIPHSR**C**RLLANHKGTLVMSSKHVCYGYQ
LRALEVFGRDRMAQVPPNKAGDSLLIS**H**V**C**GTRN**C**CRRT**H**LVIEPKHI**N**DERVH**C**HMVAHWMWKKSKRYGKRMAK
VRAAVQLA**C**P**H**EPK**C**LTSVRDLDVKDRFVESKPSNAIKYD   190

Figure S6

A

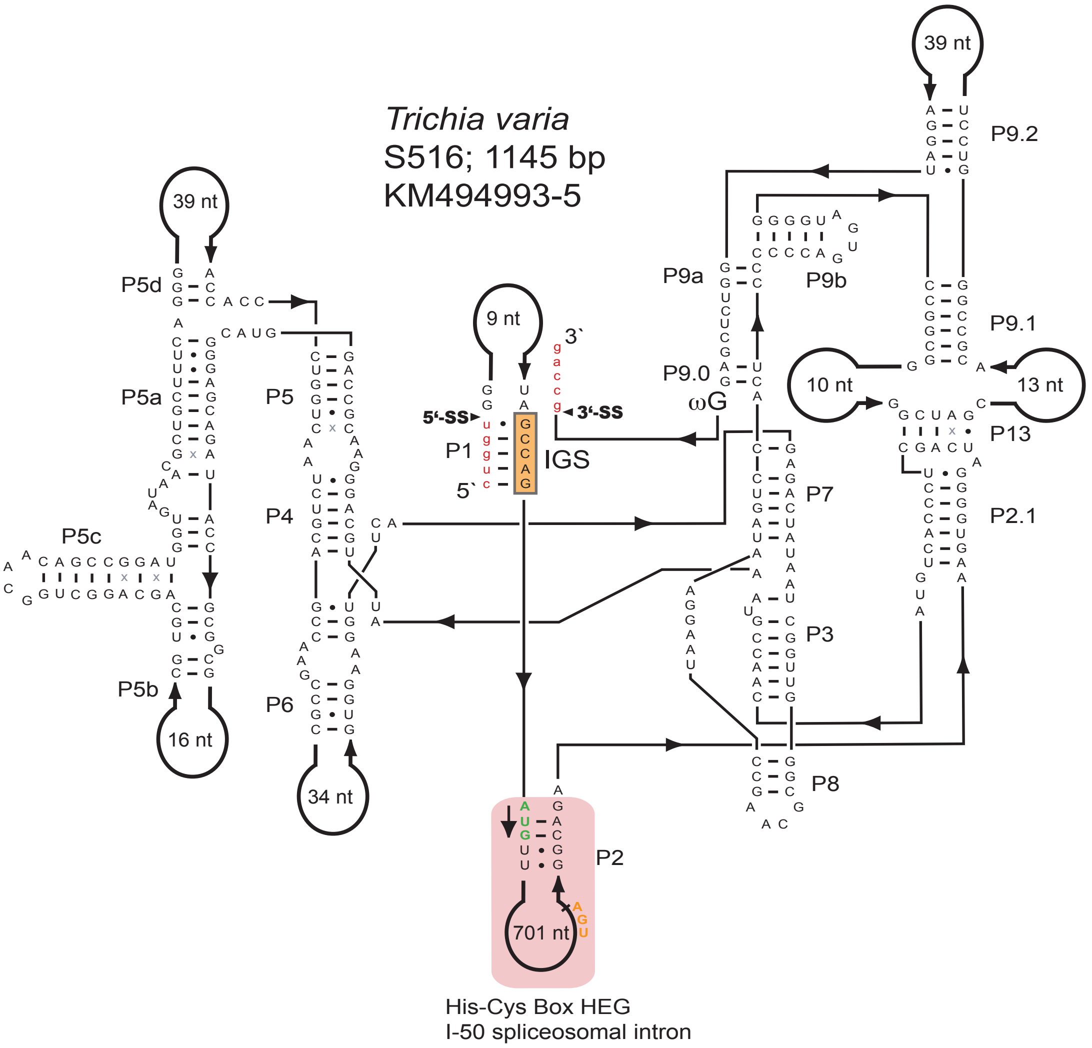

B

ATGTTTCACACGAGAAAGGCCCACAACAAGTCCAACAAAAGCCACCTATCGTTCCTAGACGAGCCGGAGGCACAC
CTTATAGAATATATAAGGGAGCTGAAGGTGTGTGGCCGAGTAATTGGGAGT**GTAGTGGGGCGGTGGCTTTGCGAG**
GTGCGTGGTACTAACACCCCGTCCAGGCTCGCCTTGTGGATGGCACATATCATGCGGGCCTTTGCCAAGGCTCGC
CCTGTGAGATGCTGCGGTCCCTGAAAGGCAGGCTGATGACTACTCGGTTGCGGGACCACAAGCCAAGCGTGGACA
TCGCTTATGGGTATCAGTTGATAGCCCTGGAGAAGTTTGGGATCGGAATTATGCGGGAGGTTGCGCCGTCTAAGG
ACAGGGACTCGATTACAATATCACATTTGTGCGGGACCCTGCTCTGCTGCAGTGCGGATCACCTGGTGTTGGAAC
CCAAATGGCTGAACGACGAGCGGACTTGCTGCCACTTCGTACTGCTTAGTGCTTTTAAGCACGGCGGGTTAGAGG
CAGTGACCGCCGTTCTGGAGGCCGGCGGGTGCCCGCATACACCTCAGTGTGGCTCCATTGCTGTTAATAAGATAG
TACGCTTCCCACCACATGCTCTGTACCCCGATATAGCCCTACCTCCCCCGACGGAGGAGGAG**AATAAA**AAATATC
AATAC**TGA**

C

MMFHTRKAHNKSNKSHLSFLDEPEAHLIEYIRELKVCGRVIGSARLVDGTYHAGLCQGSP**C**EMLRSLKGRLMTTR
LRDHKPSVDIAYGYQLIALEKFGIGIMREVAPSKDRDSITIS**H**L**C**GTLL**C**CSAD**H**LVLEPKWL**N**DERTC**C**HFVLL
SAFKHGGLEAVTAVLEAGG**C**P**H**TPQ**C**GSIAVNKIVRFPPHALYPDIALPPPTEEENKKYQY 210

Figure S7

Supplementary Figure S8

Direct repeat features in S516 intron segments

P1:

Comatricha anastomosans - JQ031961

```
1: AGGGGCAGATGGGAAGCGAGCGGGG----ATTTCATGGTCCCCC---GCTTGAAAGCGGCAAGGAAGC-GGGGAGGAA
2: AGGGGTGGACGGGAAGCGAGCGGGG----ATTTCATGGTCCCCC---GCTTGAAAGCGGCAAGGAAGCAGGGGAGGAA
3: --GGGTGGACGGGAAGCGAGCGGGGGGGGATCTCACGGTCCCCCCCCGCTTGAAAGCGGCAAGGAAACAGGGGGGGAA
4: AGGGGTGGACAAAAAGCGAGCGGGGG---ATCTCATGTTCTCCC---GCTTGAAAGCGGCAAGGAAGCAGGGGGGGAA
```

```
1: TCTCCCC-GC-TTCCCCACTGCGTTCCCAGATGCCCCTG
2: TCTCCCCTGCCTTCCCCACTGCGTTCCCAGATGCCCCTG
3: TCTTCCCTGCCTTCC-TACTGCGTTCCCAGATGCCCCTG
4: TCTCCCCTGCCTTCCCCACTGCGTTCTCGGACACCCCTG
```

Copy no: 4
Motif size: 107 nt – 114 nt

Comatricha pseudoalpina - DQ903673

```
1: AGGGGGGCGGATGGGAAGCGGGCGGGG--ATTTCATGATCCCCC-GCTTGAAAGCGGCAAGGAAGCGTGGGG--TCA
2: AGGGG--CGGACGGGAAGCGGGCGGGGGGATCTCATGTTCCTCCCGCTTGAAAGCGGCAAGGAAGCGACGGG-GCCA
3: GGGGG--TGGACGGGAAGCGGGCGGGGG-ATTTCATGTTCCCCCCGCTCGAAAGCGGCAAGGAAGCGAGGGG-CCCA
4: AGGGG--TGGACGAGAAGCGGCGGGGG--ATTTCATGTCCCCCC-GCTTGAAAGCGGCAAGGAAGCGGGGGGGGTAA
```

```
1: TTCCCCC-----GCTTCCCCACTGCGTTCCCAGATGTCCCAG
2: TTCCCC----TAGCTTCCCCACTGCGTTCCCAGATG-CCTCT
3: TTACCCC----TGCTTCCCCACTGCGTTCCCCGATACCCCTG
4: TCTCCCCCCCTGCCTTCCCCACTGCCTTCTCGGACGCCCCTG
```

Copy no: 4
Motif size: 109 nt – 114 nt

P2:

Comatricha nigricapillitia - AY643824

```
1: ACAA
2: ACAA
3: ACAA
4: ACAA
```

Copy no: 4
Motif size: 4 nt

Lamproderma album - JQ031971

```
1: ACCGATCTCACGCCCAACGAACCAAGCATCCTCGGGGAGATGATGGTTGCCACACGGAACGACGCACCCTGGTCA
2: ACCGATCTCGCGCCCAACGAACCAAGCATCCTCGGGGAGATGATGGTCGCCACATGGAACGACGCACCCTGGTCA

1: GGTGCCGAGTTCCGGGCTCGATGC-----GATCTCTG-CCCTCGGGGTGGCAGGGAACGCGCTTGTGGATCGACA
2: GGTGCCGAGTTCCAGGCTCGATGCACAGCGATCTCTGGCCCTCGGGGTGGCAGGGAACCCGCTCGTGGGTCGACG

1: GAAGGCGTGGGGGGAGTCATGAAGCGGAACGAGCCCTCGGGGCGGCTCTGGAGGCGGACGGGCAAGGTGT-GCAGG
2: GAAGGCGTGGGGAGAACCCATAAGCGGAACGAGCCCTCGGGGTGGCTCTGGAAGCGGACGGGCGAGGTGTTGCAGG
```

Copy no: 2
Motif size: 219 and 226 nt

Lamproderma columbinum - HQ687204

```
Motif I:
1: GACCAATCGTGATTCTTGTT
2: GACCAATCGTGATTCTTGTT
3: GACCAATCGTGATTCTTGTT
4: GACCA-TCGTGATTCTTGTT
5: GACCAATCGTGATTCTTGTT
```

Copy no: 5
Motif size: 19 and 20 nt

```
Motif II:
1: GACTAAAAGGGAGGGGGTAAAT
2: GACTAAA-GGAAGGG--TAAAT
3: GACTAAA-GGGAGGGGGTAA-T
```

Copy no: 3
Motif size: 19 - 22 nt

Licea parasitica - JX481297

```
1:  GGAGGGATACCTTTTCCGCGGCA      I
2:  GGAGGGATACCTTTTCCGCGGCA      I
3:  GGAGGGATACCTTTTCCGCGGCA      I
4:  GGAGGGATACCTTTTCCGCGGCA      I
5:  GGAGAGGCACCTTTTCCGCGGCA      II
6:  GGAGGGACACCTTTTCCGCGGCA      III
7:  GGAGGGACACCTCTTCCGCGGCA      IV
8:  GGAGAGGCACCTTTTCCGCGGCA      II
9:  GGAGAGGCACCTTTTCCGCGGCA      II
10: GGAGGGATACCTTTTCCGCGGCA      I
11: GGAGGGGCACCTCTTCCGCGGCA      V
12: GGAGAGGCACCTCTTCCGCGGCA      VI
13: GGAGAGGCACCTTTTCCGCGGCA      II
14: GGAGAGGCACCTTTTCCGCGGCA      II
15: GGAGGGATACCTTTTCCGCGGCA      I
16: GGAGGGACACCTCTTCCGCGGCA      IV
17: GGAGGGACACCTCTTCCGCGGCA      IV
18: GGAGGGACACCTCTTCCGCGGCA      IV
19: GGAGGGACACCTCTTCCGCGGCA      IV
20: GGAGGGACACCTCTTCCGCGGCA      IV
21: GGAGGGACACCTCTTCCGCGGCA      IV
22: GGAGGGACACCTCTTCCGCGGCA      IV
23: GGAGGGACACCTCTTCCGCGGCA      IV
24: GGAGGGACACCTCTTCCGCGGCA      IV
25: GGAGAGGTACCTCTTCCGCGGCT      VII
```

Copy no: 25
Motif size: 23 nt

Trichia varia – KM494996

```
1: AAAGGCCCGCCTTT
2: AAAGGCCCGCCTAT
```

Copy no: 2
Motif size: 14 nt

P5:

Comatricha pseudoalpina - DQ903673

```
1: GGGTGAGATCGATAAAGGTTTGGGCTATTAACGCCTGCGTGCCTGCTTCTTTCAA-GCCA
2: GGGCGAGATCGATAAAGGTTTGGGCTATTAACGCCTGCGTACCTGCTTCTTTCAAAGCCA
```

Copy no: 2
Motif size: 59 and 60 nt

P6:

Elaeomyxa cerifera - JQ031967

<u>Motif I:</u>

```
1: TAGGGTTACGC
2: TAGAGTTACGT
3: TAGGGTTACGC
4: CAGGTTTACGC
```

Copy no: 4
Motif size: 11 nt

<u>Motif II:</u>

```
1: TAACCCTAGA
2: TAACCCTAGA
3: TAACCCTAGG
4: AAACCTTAGC
5: TGACCCTAGG
6: TAACCCTAGG
```

Copy no: 6
Motif size: 10 nt

<u>Motif III:</u>

```
1: CAACCCTATGCAAAATCTTATGTTTCGGTGCCTCTAGGCCCCCGCGGTGAAAACCATGGTGGTTTGGTAAAAATCGAAGC
2: CAACC-TATGCAAAACCTTATGTTTCGGTGCCTCTGGGCCACAGTGGTGAAAACTATAGTGGTTTGGTAAAAATCGAAGC
```

Copy no: 2
Motif size: 79 and 80 nt

P8:

Colloderma oculatum - JQ031959

```
1: CCCCGCCCGTCGACCCTTGGCCCCCCCCTCGTCATGAGGGGGGGGTCTCGGCGAGGGTAGGGGTCCTGCCCGTCG
2: TCCTGCCCGTCGACCCTTGGCCTGCCCCTCGTCATGAGGGGGGGGTCTCGGCGAGGGT----GTCTGCCCTGTCG
```

```
1: ACCCTTGGCCCGCCCTCGTCATGAGGGGGTGTCTCGGCGAGGG-TGGGG
2: ACCCTTGGCCTGCCCTCGTCGTGAGGGGGGGTCTCGGCGAGGGGTGGGG
```

Copy no: 2
Motif size: 120 and 123 nt

Lamproderma columbinum HQ687196

<u>Motif I:</u>

```
1: CTTT-AAAGGAACTG
2: CTTT-AAGGGA-CTC
3: CTTT-AAGGGA-CTG
4: CTTT-AAGGGA--TC
5: CTTTGAAGAGT-CAG
```

Copy no: 5
Motif size: 11 - 14 nt

<u>Motif II:</u>

```
GGCTTTAGTC
GGTTTTAGTC
```

Copy no: 2
Motif size: 10 nt

P9:

Lamproderma columbinum - HQ687204

```
1: AAAGCAGTCAAACCAGTTAAAAGCAGTC
2: AATCCAGTCAAACCAGTTAAAAGCAGGC
```

Copy no: 2
Motif size: 28 nt

Lamproderma columbinum - HQ687200

```
1: AAACCAGTT
2: AAACCAGTC
3: AAACTACGC
```

Copy no: 3
Motif size: 9 nt

Trichia varia S516 Group I intron with and without HEG

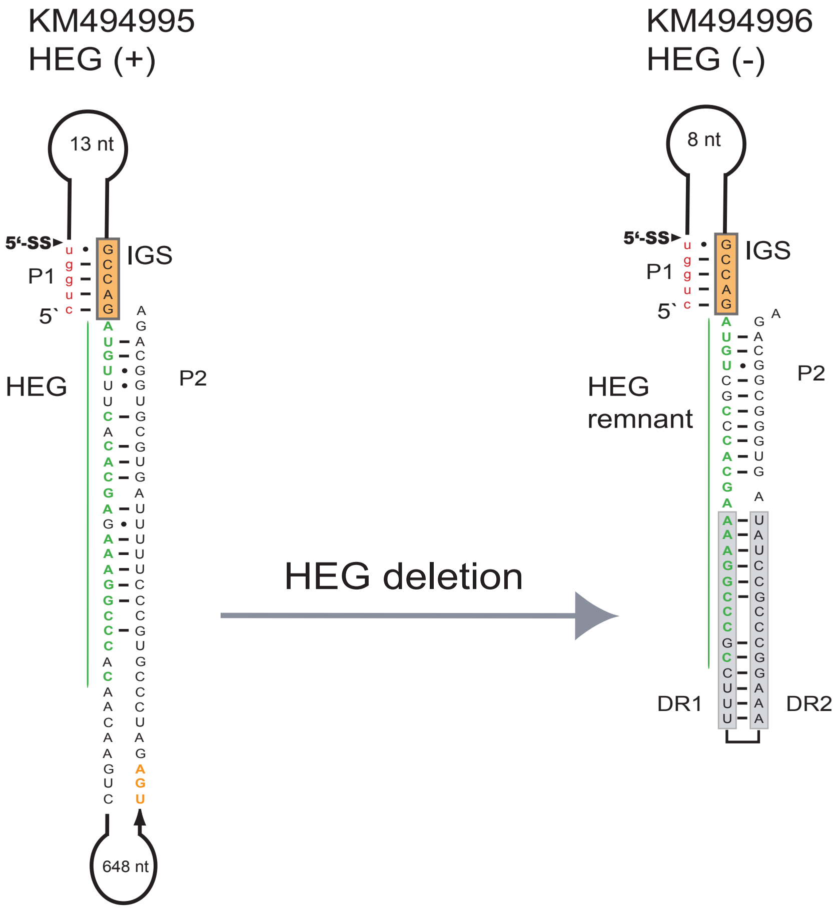

Figure S9

Table S1

Key features of 75 nucleolar group I introns at position S516 in myxomycetes.

Host species	Strain/ isolate	S516	Acc. Number	Insert
**Myxomycetes**				
**Order: Physarales; Family: Didymiceae**				
Diderma alpinum	Fr-K12	534 bp	ON155994	-
Diderma niveum	Fr-K10	496 bp	HE614615	-
Diderma niveum	It-K66	496 bp	HE614616	-
Diderma niveum	Uk-K79	496 bp	HE614617	-
Diderma soundersii	Mx-K30	482 bp	MZ313549	-
Lepidoderma crustaceum	It-K62	474 bp	HE614619	-
Lepidoderma tigrinum	AMFD192	501 bp	DQ903678	-
**Order: Physarales; Family: Physarceae**				
Badhamia gracilis	Az4-1	428 bp	AJ555451	-
Fuligo leviderma	AMFD130	402 bp	DQ903676	-
Fuligo septica	NY-1	534 bp	AJ584697	-
Fuligo septica	HMJAU1009	538 bp	MF983561	-
Fuligo septica	unknown	534bp	AJ555452	-
Physarum flavicomum	UFF6	>493 bp	AJ555453/4	-
Physarum notabile	LE47491	574 bp	JX035988	-
Physarum sp.	LE255721	441 bp	JX035984	-
Physarum sp.	AMFD-2018a	445 bp	MK336180	-
**Order: Stemonitales; Family: Stemonitaceae**				
Comatricha anastomosans	Now12905	1411 bp	JQ031961	DR-P1
Comatricha nigricapillitia	MM21077	489 bp	DQ903685	-
Comatricha nigricapillitia	Isolate 3	564 bp	AY643824	DR-P2
Comatricha pseudoalpina	MM23892	1471 bp	DQ903673	DR-P1; DR-P5c
Colloderma oculatum	HS2885	719 bp	JQ031959	DR-P8
Colloderma robustum	AMFD270	510 bp	JQ031960	-
Diacheopsis pauxilla	MM29883	516 bp	JQ031966	-
Elaeomyxa cerifera	MM24498	725 bp	JQ031967	DR-P6
Lamproderma acanthosporum	MM36058	536 bp	JQ031968	-
Lamproderma aeneum	MM36255	497 bp	JQ031969	-
Lamproderma aeneum	AK06013	>490 bp	JQ031970	-
Lamproderma album	MM37151	993 bp	JQ031971	DR-P2
Lamproderma columbinum	Isolate F2	821 bp	HQ687204	DR-P2; DR-P9.2
Lamproderma columbinum	Isolate 106	885 bp	HQ687196	DR-P8
Lamproderma columbinum	Isolate 90	699 bp	HQ687198	-
Lamproderma columbinum	Isolate 94	650 bp	HQ687199	-
Lamproderma columbinum	Isolate 63b	594 bp	HQ687197	-
Lamproderma columbinum	Isolate 132	650 bp	HQ687200	DR-P9.2
Lamproderma echinosporum	AMFD136	510 bp	JQ031980	-
Lamproderma echinosporum	AK06016	509 bp	JQ031979	-
Lamproderma pseudomaculatum	MM37354	1082 bp	JQ031985	HEG-P2; I-58
Lamproderma puncticulatum	Isolate 172	623 bp	HQ687194	-
Lamproderma sp.	AMFD-2011a	449 bp	JQ031994	-
Macbrideola oblonga	unknown	462 bp	DQ903682	-
Meriderma aggregarum	AMFD135	484 bp	DQ903669	-
Meriderma cribrarioides	MM37106	489 bp	JQ032000	-
Stemonitis fusca	MM39482	460 bp	MK041079	-
Stemonitis fusca	AMFD519	482 bp	MK041078	-
**Order: Liceales; Family: Liceaceae**				
Licea parasitica	AMFD341	1149 bp	JX481297	DR-P2
**Order: Liceales; Family: Reticulariaceae**				
Reticularia lycoperdon	AMFD262	494 bp	JX481311	-

**Order: Trichiales; Family: Trichiaceae**				
Arcyria globosa	AMFD252	459 bp	JX481282	-
Trichia decipiens	unknown	457 bp	FJ810499	-
Trichia varia	LE254838	443 bp	KM495030	-
Trichia varia	MdH-FR1210005	443 bp	KM495011	-
Trichia varia	sc22520	443 bp	KM495025	-
Trichia varia	sc27667c2	443 bp	KM495027	-
Trichia varia	sc27556c1	443 bp	KM495026	-
Trichia varia	sc27860c4	443 bp	KM495022	-
Trichia varia	sc27839	443 bp	KM495010	-
Trichia varia	sc27742	443 bp	KM495009	-
Trichia varia	sc22409	443 bp	KM494999	-
Trichia varia	AMDF451	443 bp	KM495017	-
Trichia varia	sc27664c1	443 bp	KM495008	-
Trichia varia	JVR848	482 bp	KM494996	DR-P2
Trichia varia	sc27850c2	443 bp	KM495029	-
Trichia varia	sc27507	443 bp	KM495023	-
Trichia varia	sc27745	443 bp	KM495012	-
Trichia varia	sc27648c1	443 bp	KM495024	-
Trichia varia	sc27850c1	443 bp	KM495021	-
Trichia varia	sc22370	1145 bp	KM494993	HEG-P2; I-50
Trichia varia	sc27772c3	443 bp	KM495013	-
Trichia varia	sc22556	443 bp	KM495007	-
Trichia varia	sc27697	443 bp	KM495006	-
Trichia varia	sc22517	443 bp	KM495005	-
Trichia varia	LE256579	443 bp	KM495031	-
Trichia varia	LE259461	1145 bp	KM494995	HEG-P2; I-50
Trichia varia	LE259268	1145 bp	KM494994	HEG-P2; I-50
**Order: Trichiales; Family: Dianemataceae**				
Dianema nivale	MM29888	538 bp	JX481289	-
**Order: Echinosteliales; Family: Echinosteliaceae**				
Echinostelium coelocephalum	ATCC MYA-2984	1136 bp	AY842033	HEG-P2; I-59

Table S2
Key features of direct repeat arrays in myxomycete S516 introns.

DR region	S516 intron [1]	Motif [2]	Copy no	Heterogenity [2]
P1	*Comatricha anastomosans* - JQ031961	ca 110 nt	4	+
	Comatricha pseudoalpina - DQ903673	ca 110 nt	4	+
P2	*Comatricha nigricapillitia* - AY643824	4 nt	4	-
	Lamproderma album - JQ031971	ca 220 nt	2	+
	Lamproderma columbinum - HQ687204	ca 20 nt	5	+
		ca 40 nt	3	+
	Licea parasitica - JX481297	23 nt	25	+
	Trichia varia - KM494996	14 nt	2	+
P5	*Comatricha pseudoalpina* - DQ903673	ca 60 nt	2	+
P6	*Elaeomyxa cerifera* - JQ031967	11 nt	4	+
		10 nt	6	+
		Ca 80 nt	2	+
P8	*Colloderma oculatum* - JQ031959	ca 120 nt	2	+
	Lamproderma columbinum - HQ687196	ca 12 nt	5	+
		10 nt	2	+
P9	*Lamproderma columbinum* - HQ687204	28 nt	2	+
	Lamproderma columbinum - HQ687200	9	3	+

Notes:
1) More detailed information about the S516 introns is provided in Tabel S1.
2) Motif sequences and heterogeneity are presented in Figure S8.
